# Triangulating evidence for genetic and environmental components of associations between parental behaviours and aggressive behaviour in children

**DOI:** 10.1038/s41467-026-75095-5

**Published:** 2026-07-06

**Authors:** Joanna K. Bright, Yasmin I. Ahmadzadeh, Olakunle Oginni, Essi Viding, Leslie D. Leve, Jenae M. Neiderhiser, Daniel S. Shaw, Misaki N. Natsuaki, Jody M. Ganiban, Eivind Ystrom, Tom A. McAdams

**Affiliations:** 1 Social, Genetic & Developmental Psychiatry Centre, Kings College London, London, UK; 2 The Wolfson Centre for Young People’s Mental Health and Centre for Neuropsychiatric Genetics and Genomics, University of Cardiff, Cardiff, UK; 3 Division of Psychology and Language Sciences, University College London, London, UK; 4 Department of Education, University of Oregon, Eugene, OR USA; 5 Department of Psychology, The Pennsylvania State University, University Park, PA, USA; 6 Department of Psychology, University of Pittsburgh, Pittsburgh, PA USA; 7 Department of Psychology, University of California, Riverside, CA USA; 8 Department of Psychological and Brain Sciences, George Washington University, Washington, DC USA; 9 PROMENTA Research Center, University of Oslo, Oslo, Norway; 10 Department of Child Health and Development, Norwegian Institute of Public Health, Oslo, Norway

**Keywords:** Human behaviour, Genetics

## Abstract

Parenting behaviours are often linked to childhood aggression, but many studies fail to account for shared genetics or test directionality. Using family-based designs, we control for genetic confounding and examined bidirectional effects across two samples. First, we apply a children-of-twins/siblings design in the Norwegian Mother Father and Child Cohort Study which uses genetic relatedness between family members to separate genetic and environmental components of parent-child associations. Second, we use longitudinal models in the USA-based Early Growth and Development Study, an adoption sample where parents and child are not genetically related, enabling estimation of parent-child associations without genetic confounding.

Across both studies, we find evidence for genetic and environmental transmission between parenting and child aggression. We show significant child-to-parent effects on inconsistent discipline in both samples. In the children-of-twins analysis, the association between positive parenting and child aggression is shown to be due to shared genetics, where no statistically significant association is observed in the adoption sample. These findings build on the growing body of literature showing genetic influences on both aggression and parenting behaviours, whilst underscoring the value of triangulation.

## Abstract (web summary)

The authors use family-based design to show evidence for child-to-parent effects on inconsistent discipline as well as for the contribution of shared genetics to associations between aggressive behaviour in children and parenting.

## Introduction

Child aggression is commonly seen across many psychopathology profiles, co-occurring with a broad spectrum of behavioural, emotional and social difficulties^1^. Aggressive/disruptive behaviour in children has been linked to poorer outcomes later in life, such as peer rejection^2^ and poorer general health and difficulties engaging with education^3^. Aggressive expression of frustration via ‘tantrums’ or lashing out is a normal human response shown by most children^4,5^. Typically, physical aggression peaks in frequency between 2-4 years old, after which aggressive behaviours decrease, as children learn more socially acceptable ways of expressing their frustration and anger^5^, and self-regulatory skills improve (e.g., effortful control)^6^. However, higher levels and/or persistence of child aggression (i.e., aggression that is not age normative) can lead to difficulties for the child’s friendships^2^ and the parent-child relationship^7^, and predict relationship instability during adulthood^8^. It is therefore important to understand how and why high levels of child aggression develop. Identifying potential causal environmental influences on childhood aggression can help researchers pinpoint targets for intervention^9^. A demonstration of genetic influences does not preclude social or behavioural interventions but will alert researchers and clinicians to potential challenges that need to be anticipated.

Parenting style is frequently associated with aggressive outcomes in children. For example, negative parenting behaviours, such as inconsistent discipline and harsh parenting, are linked to higher levels of aggressive and antisocial behaviours in children^10,11^, beginning as early as infancy^12,13^, and showing prediction to more serious outcomes in adolescence beginning in the toddler period^14^, including prediction to adolescent violent behaviour^15^. Conversely, higher levels of positive parenting behaviours relate to lower levels of child disruptive behaviour^16^. There are many pathways that could lead to the development of these associations between parent and child, both causal and non-causal, and it is important for research examining the transmission of behaviours to consider multiple pathways when describing such a relationship.

Firstly, it is possible that associations between parenting behaviours and child aggressive behaviours exist in part because parenting styles cause aggressive behaviours and/or the reverse, whereby aggressive behaviour in children evokes particular parenting styles. Previous research suggests that some parenting behaviours associated with child aggressive and antisocial behaviour may occur in response to the child’s behaviour^17,18^. Child aggressive and antisocial behaviour has been shown to be a predictor of greater use of punitive discipline^19^ and lower levels of positive parenting behaviours^20^. Parent-to-child effects have also been shown, with timid discipline predicting worsening oppositional defiance symptoms, including aggressive behaviours^17^. Low maternal warmth, controlling behaviour and hostility have also been shown to be associated with the development of subsequent antisocial behaviour, including aggressive behaviours, in children^17,21^. Understanding the possible bidirectional influences between parent and child behaviours may help identify modifiable targets for prevention and treatment of psychopathology and relationship difficulties.

Parents are the source of both a child’s DNA and their rearing environment, and this can make it difficult to distinguish the effects of one from the other: Passive gene-environment correlation (a correlation between rearing environment and genome)^22^ may partially or entirely explain associations between a child’s environment (e.g., including the parenting they receive) and their genetically influenced traits (e.g., aggression). Genetically informed research has shown genetic confounding in parent-child transmission of conduct problems, which captures aspects of aggression^23^. When looking at the direction of causation, research has described an evocative role of children’s genetically mediated aggression and delinquency on parental criticism^24^. Similarly, research using the EGDS adoption sample has described a small evocative effect of child antisocial behaviour via the birth parents’ genotype on the adoptive parenting behaviours^25^. However, it is not yet clear whether there is genetic overlap between parenting behaviours and child aggression, wherein the genes associated with parenting behaviours in parents may also influence aggression in children.

In this paper, we make use of two family-based designs which allow for direction-of-causation analysis while controlling for passive gene-environment correlation (rGE). Family-based designs, such as children-of-twins and adoption studies, offer opportunities to parse out the potential influence of parent on child from the effects of shared genes and environments. Further, family data can control for unmeasured shared environmental influences, such as geographical or socioeconomic pressures, as families are assumed to experience these effects similarly. First, we used a multiple children-of-twin-and-siblings (MCoTS) sample taken from the Norwegian Mother, Father and Child Cohort Study (the MoBa study). For a detailed description of children-of-twins models and their extensions, we refer readers to McAdams et al.^26,27^. Briefly, the MCoTS design decomposes parent-child associations into genetic and environmental components by comparing monozygotic (MZ) and dizygotic (DZ) avuncular correlations (correlations between uncle/aunt and niece/nephew) to parent-child correlations. The children of MZ twins share 50% of their genes with their parent, and their parent’s co-twin, meaning they are as genetically related to their aunt/uncle as they are to their parent, whereas children of DZ twins share 25% of their genes with their aunt/uncle. In this way, we can assess whether associations are stronger in more genetically related individuals to consider the role of genetics in intergenerational assessments, and whether there are remaining parent-child associations once controlling for shared genetics. The MCoTS model can be further adapted to assess the direction of causation^24,28,29^. Second, we used a longitudinal parent-offspring adoption sample, the Early Growth and Development Study (EGDS). In parent-offspring adoption studies, where the adoptive parents and child are not genetically related, correlations between adoptive parent traits and child traits are free from passive rGE confounding. Thus, any association between parenting and child aggression must be assumed to be environmental (i.e., either prenatal/delivery effects, an effect of parent on child, an effect of child on parent, or attributable to environmental confounding). Longitudinal adoption studies can further help to distinguish the direction of causal influences between parent and child.

The MoBa and EGDS studies have comparable measures of parenting and child aggression to distinguish between different mechanisms for the associations between parenting and the child’s aggression, including parent-on-child, child-on-parent, and genetic inheritance. Taken on their own, both samples allow for genetically informed bidirectional analyses. However, they each involve using specific samples and rely on certain assumptions. This reliance on complex methods and specific samples can raise questions about the generalisability of the results when only one method is deployed. In the present paper, we test for bidirectional effects between parenting and child aggressive behaviour using two distinct family-based, genetically informed methods in separate samples matched on age range (from 4.5- to 8-years-old). Both samples include items from the same measures of inconsistent discipline and positive parenting behaviours, as well as child aggressive behaviours. By interrogating pathways between parenting behaviours and child aggression in two different genetically informed samples, we harness the opportunity to address whether we are picking up on a generalisable relationship or whether findings are specific to a sample or method.

## Results

In this study, we made use of cross-sectional drawn from a large birth cohort, the Norwegian Mother and Baby Study (MoBa), and longitudinal data from a US adoption study, the Early Growth and Development Study (EGDS). We first ran an extended family design model in MoBa to estimate the direction of effects between parenting and childhood aggression while accounting for genetic confounding. We employed the Multiple Children of Twins/Siblings (MCoTS) model, which decomposes intergenerational associations between parenting and child aggressive behaviours into genetic and environmental components (Supplementary Fig. 1). Secondly, in EGDS, we used longitudinal designs to estimate the direction of effects between parenting and child aggression while accounting for genetic confounding using adoptive families.

See Supplementary Table 1 for a full overview of pedigree structures and Supplementary Table 2 for descriptive statistics for the measures of interest in MoBa. We created latent factors for each of the mother and child measures for use in the MCoTS models. See Supplementary Table 3 for parameter estimates for each factor and its loadings. Fit statistics showed each factor had good fit to the data (See Supplementary Table 4). In MoBa, the positive parenting factor showed a negative correlation with child aggression (estimated at *− *0.11, *р* = 0.000; Supplementary Table 5) and inconsistent discipline positively correlated with child aggression (estimated at 0.23, *р* = 0.000; Supplementary Table 5).

See Supplementary Table 6 for sample size and descriptive statistics for the EGDS sample. Supplementary Table 7 shows the pairwise correlations between variables. Inconsistent discipline and child aggression were moderately correlated across each time point (significant correlations ranged from 0.15 to 0.34, *р* = 0.000-0.004). Positive parenting and child aggression was only significantly correlated at age 4.5 years (*r* = − 0.18, *р* = 0.000).

To aid comparison across all analyses, Table 1 provides a qualitative summary of the key results from each sample, for the analyses with inconsistent discipline and child aggression, and positive parenting and child aggression.

**Table 1:** Qualitative summary of outputs from MCoTS and longitudinal models

Sample	Inconsistent Discipline & Child Aggression	Positive Parenting & Child Aggression
MCoTS (MoBa)	Finding: Relationship partially explained by shared genetics. Remaining phenotypic relationship driven by child-to-parent effect Implication*:* Beyond the effect of shared genetic influences, inconsistent discipline can be understood as a consequence of child aggression	Finding: Relationship wholly explained by shared genetics Implication: All the associations between positive parenting and reduced child aggression reflect shared genetic influences between parents and children
Adoption (EGDS)	Finding: Significant across- and within-time effects from child-to-parent Implication*:* In the absence of shared genetic influences (parents and children in this sample are not genetically related), inconsistent discipline can be understood as a consequence of child aggression	Finding: No significant effects of parent-to-child or child-to-parent Implication*:* There is no evidence for an association between positive parenting and aggression in children in an adoptive sample where parent and children are not genetically related.

### Inconsistent discipline and childhood aggression: MoBa birth cohort

The correlation between maternal inconsistent discipline and child aggression was 0.22, and we found no significant influence of shared environment (C paths; See Supplementary Fig. 2 for full model specifications), and so we dropped these paths from the final MCoTS model (Supplementary Fig. 3). Fifty-three percent of the mother-child correlation was explained by environmental factors and 47% was attributable to genetic correlation (See Supplementary Table 8 for summary of genetic and non-genetic contributions). There was a remaining phenotypic relationship (*p* path estimated at 0.10; see Supplementary Fig. 3) after adjusting for genetic correlation between parent and child. Table 2 summarises all path estimates in the final MCoTS models, and Supplementary Table 9 details comparative model fit statistics for all models tested.

**Table 2:** Structural equation model ACE components from MCoTS models

	A1	E1	A1'	A2	E2	p	m (parent-to-child)	n (child-to-parent)
Inconsistent Discipline†	0.34 (0.30, 0.38)	0.66 (0.56, 0.70)	0.31 (0.10, 0.55)	0.17 (0.06, 0.35)	0.38 (0.28,0.50)	0.10 (0.06, 0.15)	–	–
Inconsistent Discipline (bidirectional)	0.28 (0.25, 0.35)	0.72 (0.72, 0.75)	0.20 (0.15, 0.20)	0.35 (0.31, 0.37)	0.45 (0.43, 0.48)	–	0	0.11 (0.11, 0.11)
Positive Parenting	0.18 (0.05, 0.41)	0.82 (0.59, 0.95)	0.16 (0.06, 0.49)	0.40 (0.19, 0.49)	0.44 (0.29, 0.52)	0	–	–

Note: Standardised path estimates (95% confidence intervals). A1 = additive genetic effects on parental phenotype; E1 = nonshared environmental effects on parental phenotype; A1′ = genetic effects common to parental phenotype and offspring phenotype; A2 = additive genetic effects specific to offspring phenotype; E2 = nonshared environmental effects on offspring phenotype; *p* = remaining behavioural association; *m* = phenotypic effect of parent on offspring. *n* = phenotypic effect of offspring on parent. C1, C1’ and C2 paths (shared-environmental effects on parent & offspring phenotypes were non-significant in the full model and so were dropped, thus have not been reported here. †This model was initially run with a single phenotypic path and extended in the next model to incorporate bidirectional causality with the m and n paths.

**Fig. 1:**
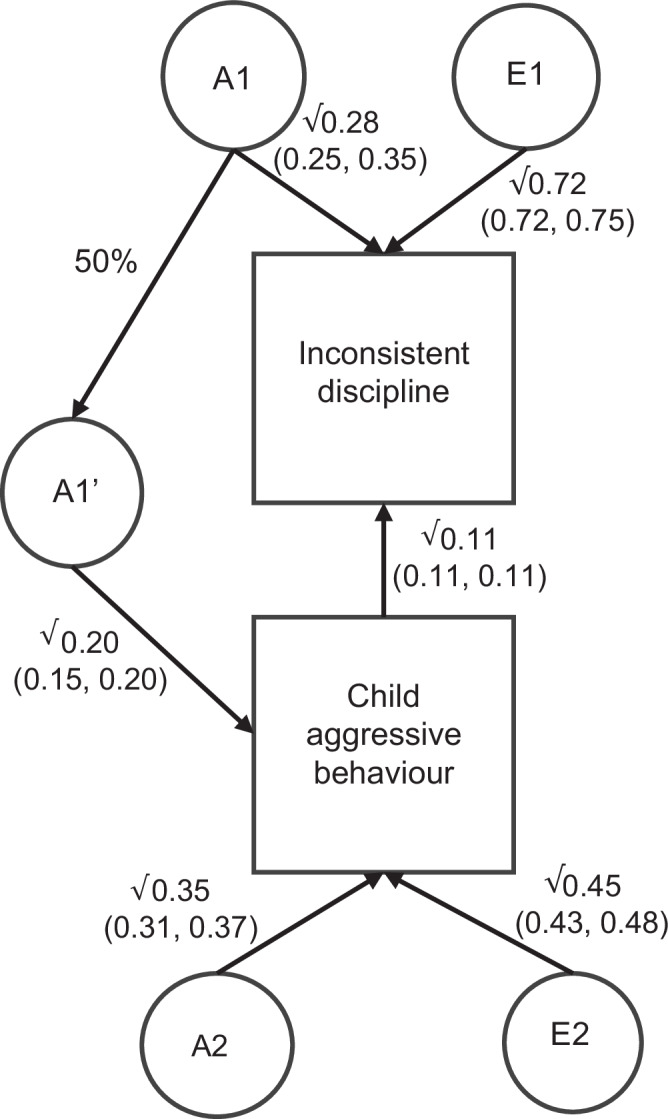
Results from the Multiple-Children-of-Twins/Siblings – Direction of Causation model for inconsistent discipline. Figure represents a partial path diagram of the best-fitting model decomposing the association between mothers’ inconsistent discipline and child aggressive behaviour (observed association estimated at 0.22). Squares denote observed variables and circles denote latent variables. A1, additive genetic effects on mother behaviour; A2, additive genetic effects specific to child behaviour; A1′, additive genetic effects common to mother and child behaviours (path from A1 to A1′ is fixed to 0.50 because offspring inherit 50% of their mother’s genes); E1/E2, unique environment effects on mother/child behaviours. Variance components are displayed in non-bold text. The residual intergenerational association from child-to-parent is displayed as a standardised path beta coefficient in bold text.

To specify the direction of causality, we tested the MCoTS-DOC model with both parent-to-child path and child-to-parent paths, and then dropping one path at a time. The best fitting model had a child-to-parent path and genetic path but no parent-to-child path (Fig. 1, comparative fit statistics in Supplementary Table 9. See Supplementary Fig. 5 for the full bidirectional model path estimates). This indicated that the direction of the environmental effect found in the MCoTS model is from the child to the mother.

### Inconsistent discipline and childhood aggression: EGDS adoption sample

**Fig. 2:**
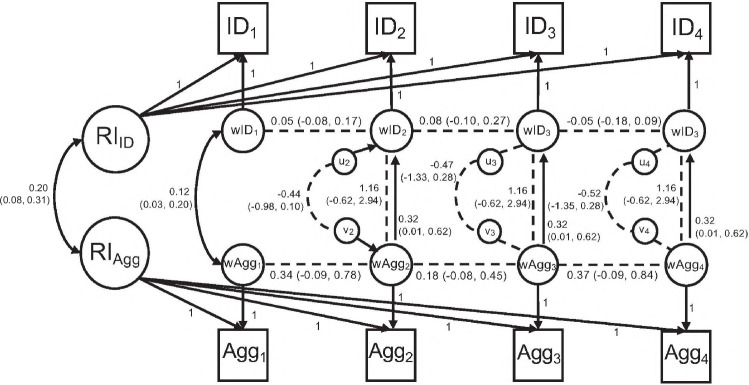
Results from the bivariate autoregressive fixed effect model for inconsistent discipline and aggression. Figure represents the best fitting model for the testing the effects of mothers’ inconsistent discipline (ID) and child aggressive behaviour (Agg) within time. Squares denote observed variables and circles denote latent variables. Note: RI = Random intercept. Significant paths are in black, non-significant paths are denoted by dotted lines.

Consistent with the MCoTS-DoC model, the bidirectional autoregressive fixed effect model for inconsistent discipline and child aggression showed a pattern of within-time child-to-parent transmission (Fig. 2). The parent-to-child paths were non-significant. The model fitted the data well (CFI = 0.998; TLI = 0.996, RMSEA = 0.020). Table 3 summarises the effect sizes of the parent-to-child and child-to-parent paths from the panel models.

**Table 3:** Parent-child effects from bidirectional and cross-lagged panel models in the adoption sample

	Parent-to-child		Child-to-parent		
Parenting dimension	Cross lags	Within-timepoint		Cross lags	Within-timepoint
Inconsistent discipline	0.07 (− 0.07, 0.18)	1.16 (− 0.62, 2.94)		**0.16 (0.01, 0.30)**	**0.32 (0.01, 0.62)**
Positive parenting	− 0.12 (− 0.23, 0.00)	− 0.66 (− 1.38,0.05)		0.01 (− 0.14, 0.15)	0.04 (− 0.24, 0.31)

Note: Standardised path estimates (95% confidence intervals). Bold text indicates significant paths.

See the Supplementary Fig. 4 for the random intercept cross-lagged panel model results, which also showed significant longitudinal cross-lagged effects in the child-to-parent direction, with non-significant paths from parent to child. Again, the model showed a good fit to the data (CFI = 0.999; TLI = 0.998, RMSEA = 0.017).

### Positive parenting and child aggression: MoBa birth cohort

**Fig. 3:**
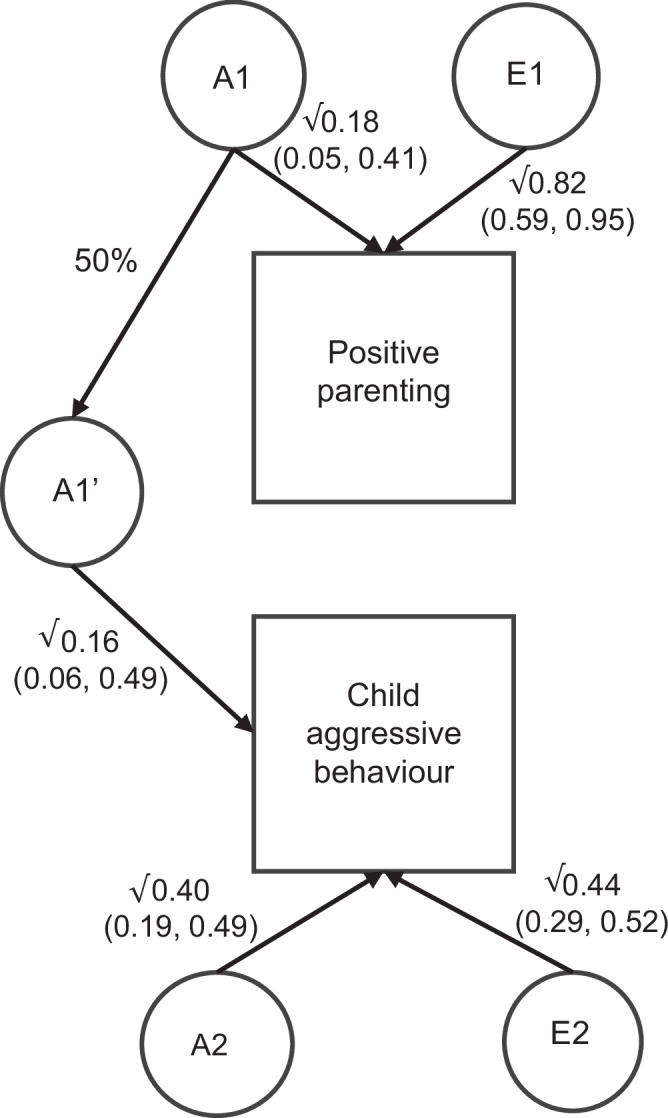
Results from the Multiple-Children-of-Twins/Siblings model for positive parenting. Figure represents a partial path diagram of the best-fitting model decomposing the association between mothers’ positive parenting and child aggressive behaviour (observed association estimated at − 0.09). Squares denote observed variables and circles denote latent variables. A1, additive genetic effects on mother behaviour; A2, additive genetic effects specific to child behaviour; A1′, additive genetic effects common to mother and child behaviours (path from A1 to A1′ is fixed to 0.50 because offspring inherit 50% of their mother’s genes); E1/E2, unique environment effects on mother/child behaviours. Variance components are displayed in non-bold text. There was no residual intergenerational association.

The correlation between positive parenting and child aggression was estimated at −0.09. Again, model fitting showed that dropping C paths from the model (see Supplementary Fig. 2 for path estimates from the full model) did not worsen model fit. Dropping the phenotypic (p) path did not worsen model fit, suggesting the association between positive parenting behaviours and lower child aggression was due to genetic overlap only. The final MCoTS partial path diagram is presented in Fig. 3 (see Table 2 for all path estimates in the final model). Given that we found no significant phenotypic association between positive parenting and child aggression, this relationship was not further explored using the MCoTS-DoC model.

### Positive parenting and child aggression: EGDS adoption sample

**Fig. 4:**
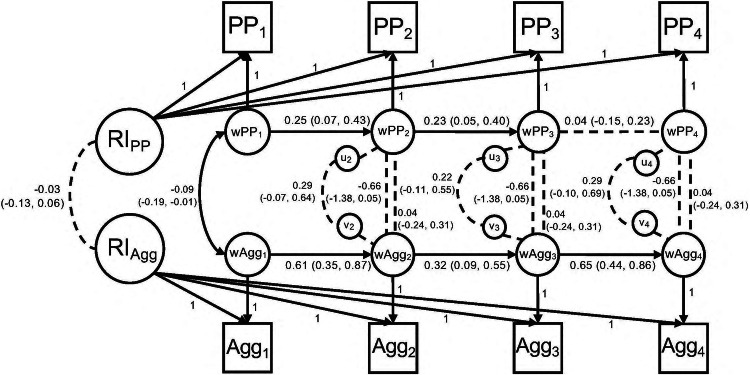
Results from the bivariate autoregressive fixed effect model for positive parenting and aggression. Figure represents the best fitting model for the testing the effects of mothers’ positive parenting (PP) and child aggressive behaviour (Agg) within time. Squares denote observed variables and circles denote latent variables. Note: RI = Random intercept. Significant paths are in black, non-significant paths are denoted by dotted lines.

The bidirectional autoregressive fixed effect model for positive parenting and child aggression showed no significant within-timepoint associations between parent and child behaviour (Fig. 4), with the model showing good fit (CFI = 1.000; TLI = 1.008, RMSEA = 0.000). Similarly, the random intercept cross-lagged model showed no significant prospective causal effects between parent and child at any timepoint (Supplementary Fig. 6, model fit CFI = 1.000; TLI = 1.000, RMSEA = 0.000). See Table 3 for all main path effect sizes from the panel models.

### Sensitivity analyses: Testing child sex-specific effects

We ran all models in samples stratified by child sex-assigned-at-birth in both the MoBa and EGDS samples. In MoBa, we found that confidence intervals for each variance component overlapped for models run in the boy-only and girl-only samples, for either dimension of parenting (see Supplementary Table 10 for ACE component estimates and Supplementary Table 11 for model fit statistics). This suggests that there are no significant sex differences in the aetiology of the parent-child associations.

In EGDS, we compared model fit for the unconstrained model (parent-to-child/child-to-parent paths estimated freely across the sexes) with the model where paths were constrained to be equal across the sexes. There was no significant difference in model fit (see Supplementary Table 12 for effect sizes of the constrained model and Supplementary Table 13 for comparative fit statistics for both models), i.e., the model where the paths were estimated as equal in boys and girls fit as well as the unconstrained model, suggesting no significant influence of sex on parent-child associations.

### Sensitivity analyses: Making use of paternal reports

Whilst only maternal reports were used in the main analyses to allow for comparison across samples, we carried out sensitivity analyses in EGDS to see whether there was a similar pattern of results when using paternal reports (available for an average of 87% of children at each wave, response rate range of 75–90% across the four waves; see Supplementary Table 14 for fit statistics). We ran sensitivity analyses using (i) paternal reports of parenting behaviours and child aggression; (ii) paternal reports of child aggression in mother-child dyads, (iii) maternal reports of child aggression in father-child dyads.

For analyses using paternal reports for both parenting and child aggression (see Supplementary Table 12 for main results), we found significant within-timepoint paths from parent-to-child and child-to-parent for the relationship between inconsistent discipline and child aggressive behaviour. However, there were no significant cross-lags. For analyses using positive parenting, there were no significant associations between paternal reports of positive parenting and child aggression either within or across time. There were no significant paths when using paternal reports of child aggression in mother-child dyads or maternal reports of child aggression in father-child dyads.

## Discussion

We used two separate genetically informative samples with the same measures of inconsistent discipline, positive parenting, and child aggression to triangulate evidence regarding the association between parenting and child traits.

For both parenting behaviours, we found evidence for genetic overlap with aggression in children in the MCoTS analyses, whereby genetic factors influencing parenting behaviours in the parent were passed on to children, in whom they influenced the degree of aggressive behaviour. Using the multiple-children-of-twins/siblings designs in the Norwegian MoBa study, we found that 47% of the association between inconsistent parenting and child aggression was explained by shared genetics. Further, the negative association between positive parenting and child aggression was completely (100%) explained by shared genetics. Together, these findings suggest that the genetic factors associated with higher levels of inconsistent discipline/lower levels of positive parenting in parents are also associated with higher levels of aggressive behaviours in children.

It was encouraging to see converging evidence across different genetically informed study designs. Where we did find a non-genetic relationship between inconsistent discipline and child aggression in the MoBa sample using a multiple-children-of-twins-and-siblings design, we also found evidence for a relationship between parent behaviour and child aggression in EGDS, an adoption sample where there was no genetic relatedness between generations. Inconsistent discipline has been previously implicated in the development of child aggression^30^; our results provide evidence for such a relationship, while highlighting that the observed association is partially attributable to gene-environment correlation (rGE). Further, our results extend on previous research by demonstrating a direction of effects, where childhood aggression drives inconsistent parenting beyond shared genetic influences. The findings of a genetic component to the aetiology of child aggression speak to previously described patterns of evocative rGE, where the child’s genotype influences their exhibition of aggressive behaviours, which actively impact their parents’ behavioural response^31^. We found no evidence for a non-genetic relationship between positive parenting and child behaviours.

Future research should investigate whether there is overlap between the genetic underpinnings of parenting dimensions and child outcomes, given that our findings suggest a shared genetic liability for child aggressive behaviours and inconsistent discipline/positive involvement from parents. There is a genetic component to an individual’s risk for aggressive behaviour or conduct-related problems. A genome-wide association study of general liability to externalising behaviours found that the resultant polygenic score predicted 2.0–2.3% of the variance in aggression, 1.2–3.1% of the variance in rule-breaking behaviour^32^ and up to 3.4% of the variance in child behaviour problems^33,34^. Intergenerational research has shown that associations between polygenic scores for parental externalising and risk-taking and child conduct problems were due to direct genetic transmission^35^. Twin designs have shown genetic influences on parenting behaviours, both at the parent level and the level of the child who is receiving the parenting^36^, suggestive evocative effects of the child genotype on the parenting they receive.

We found the strongest evidence for environmentally mediated behavioural effects when looking at the pathway between inconsistent parental discipline and child aggression. In the MCoTS analysis, we found a modest association between parent and child behaviours, with 53% of the association remaining after controlling for genetic transmission. Models which split the phenotypic path down further into parent-to-child and child-to-parent transmission showed that this behavioural transmission was driven by the child aggressive behaviours influencing inconsistent discipline.

In the adoption sample, we see a picture of the child’s aggressive behaviour predicting inconsistency in the parenting they receive in the short-term, with the cross-lagged models the same over a longer time frame. Sensitivity analyses suggest that there could also be a significant role for fathers’ inconsistent discipline influencing child aggression within the same timepoint, although these effects did not exist across timepoints. As these are non-biologically related parent-child dyads, this relationship appears to be mediated through non-genetic pathways. There is cross-sectional evidence of different pathways of maternal and paternal influence previously shown for parental criticism and problematic behaviour in older adolescents^24^, with an evocative rGE effect of child externalising on maternal criticism, whereas there was a direct environmental effect of paternal criticism. Our longitudinal findings add to the evidence base for potentially diverging aetiologies for relationships between parent and child behaviour depending on the gender of the parent. Our findings partially map onto coercion theory models, where there is a dynamic system of parents and children learning from behaviour patterns in each other in ways which may increase risk for aggressive behaviour and non-compliance in children, and also decrease parents ability to control these behaviours in their children^37^. In these cycles, children respond to parent’s disciplinary behaviours with an escalation of difficult behaviours, after which the parent backs down. Indeed, in our research, we see that parent’s discipline is more inconsistent for children exhibiting higher levels of aggressive behaviours. However, our results do not reflect the full cycle, as there were no significant paths from inconsistent parenting to later aggressive behaviours.

Across the longitudinal positive parenting models, all analyses indicated no evidence for a causal association between maternal reports of positive parenting and aggressive behaviour in children, in either the parent-to-child or child-to-parent direction. In sensitivity analyses in the adoption study, we also found no evidence for an association between paternal reports of positive parenting and child aggression. Previous literature has shown that children of mothers who displayed more positive parenting were shown to have lower levels of externalising behaviours later in life^16^; however, these authors reported their sample did not have the power to decompose genetic effects from environmental ones. Our research offers a genetic explanation for such a relationship, whilst also suggesting that there may be child-specific effects for positive parenting in non-genetically related families.

Our findings suggest that clinicians working with children should recognise that childhood aggression can drive inconsistent discipline. Thus, interventions for childhood aggression that are focused only on consistent discipline by parents may be insufficient. It may be beneficial to explore and additionally target other familial, environmental and individual factors associated with aggressive behaviours in the child. This research hints at potential causal links for parent-child behaviour patterns and evidence for the role of genetics in associations. However, measures of single domains of parent and child behaviours do not capture the complex nature of everyday parenting that interventions and clinical practice must consider. Hence, more research needs to be done to build a holistic picture of how day-to-day parenting interactions develop, and to disentangle the genetic and causal links between various domains of parenting and aggressive behaviour in children.

In this study, we triangulated across two different family-based study samples applying distinct analytical methods to the same measures of parent and child behaviours. This gives us a rare opportunity to clarify the role of genetics and environment in the aetiology of the parent-child relationship. Through multiple statistical designs which capture genetic and bidirectional relationships, we found converging evidence for both genetic and behavioural transmission impacting the relationship between parenting styles and child aggressive behaviours.

Still, there were limitations to these designs. The samples included the same subscales, however the EGDS dataset included all items of child aggression included in the CBCL, whereas MoBa included a smaller subsection of the aggressive behaviour subscale. Therefore, the subscale as measured by EGDS may be capturing a broader spectrum of aggression-related behaviours to those described in the MoBa dataset. However, we did still find converging evidence for the relationship between parent and child, suggesting the truncated versions of the subscales in MoBa were capturing the same patterns of behaviour as the full scales in EGDS. Further, while the subscale is named as an “aggression” subscale, it captures both physical aggression and more reactive types of externalising symptoms. This may have implications for the real-world interpretation of these findings, and future research could consider testing the replicability of these findings with items focused more purely on physical aggression. Whilst the measures used by both samples are rigorously validated, these subscales may only capture rather narrow dimensions of parenting and child behaviours. Therefore, used alone, they may not fully translate to clinical practice or parent-child interventions. For example, the APQ is a short measure, with only 6 items, which may limit how well it captures complex parenting dynamics. It is important for future research to continue the triangulation approach presented here with methods that can incorporate more in-depth assessments of parenting and child behaviours (e.g., observations of parenting, teacher and youth reports of child behaviour), or use mixed methods approaches which are able to incorporate more nuanced aspects of parent-child relationships.

To allow for comparison across samples, in our main analyses, we only included maternal reports of parent and child behaviours, as MoBa did not have paternal reports. Whilst we were able to run sensitivity analyses using paternal data in EGDS, it is a limitation that we cannot triangulate these findings with MoBa. Research has shown differential parent-child associations dependent on the gender of the parent^7,38^, which indeed was what we found in EGDS, where there were significant parent-to-child within time paths for inconsistent discipline. In EGDS sensitivity analyses, we compared paternal reports of child aggression in mother-child dyads and vice versa. In these analyses, we found no evidence of significant parent-to-child or child-to-parent paths, which could suggest that using the same reporter for both parent and child behaviours led to inflated associations. It is known that using the same reporters for both parent and child reports may bias responses^39^, and so the inclusion of multiple reporters for each observation of behaviour would allow researchers to control for some of these biases. Alternatively, mothers and fathers may pick up different aspects of parenting and child aggression in ways that are unrelated. For example, fathers may rate children as less aggressive than mothers, or may report their parenting as less consistent, which may reduce associations with mother-reported variables. We note that previous work in our group^33^ has shown that a polygenic score for externalising is as predictive of parent-reported child externalising behaviour as it is of child-reported and teacher-reported externalising, independently of parental indirect genetic effects, even though parent, teacher and child-report externalising are only moderately correlated. This demonstrates that regardless of the reporter, measures of externalising do index a child’s genetically influenced behaviour; thus, we can assume that parent-report is not all bias and is truly reflective of a child’s behaviour.

Further, by including only one parent in our MCoTS analyses, we could not control for the possible effects of assortative mating, the phenomenon where people choose partners who are like them for specific behaviours, i.e., are “phenotypically similar”. The implications of assortative mating for decomposing intergenerational relationships are complex, with alternative explanations for coparent similarities such as partners being alike on social or cultural factors, or become more phenotypically similar as they spend more time together^27^. There is evidence for assortative mating on aggressive or violent behaviours^40^, thus future research could incorporate coparent data and/or observational measures of parent-child interaction into MCoTS models to elucidate potential biases from assortative mating on the genetic and environmental components of the relationship between parenting behaviours and child aggression. That being said, assortative mating does not similarly impact the adoption design: in adoption studies, associations between adoptive parent and child remain attributable to non-genetic causes regardless of whether birth or adoptive parents mate selectively. That our MCoTS and EGDS analyses arrive at the same conclusions would suggest that assortative mating is not biasing our findings.

The influence of genetics will vary across different contexts or cultures where environmental pressures exert more or less influence on the expression of a trait. Both the EGDS and MoBa samples are skewed to higher levels of socioeconomic advantage^41,42^. Research has shown that shared environment has more of an influence on the aetiology of conduct related problems in young people experiencing higher levels of disadvantage, in contrast with genetics playing a larger role for those in more advantaged contexts^43–45^. It is possible that by using samples with an overrepresentation of more advantaged families, we may be missing potentially causal pathways between parent and child that exist for samples with lower socioeconomic backgrounds, preventing us from identifying targets for intervention where they may most be needed. Whilst both samples are similarly skewed to higher socioeconomic advantage, they are also from different countries, with different cultural and societal factors. Norway and the US may have slightly different cultural norms when it comes to child aggression and parenting practices, and these norms could impact how parents responded to the measures included here. However, the fact that results and conclusions across these two samples were the same for two distinct forms of parenting strongly suggests that these differences in norms do not translate to differences in mechanisms underlying parent-child associations in North American and Norwegian contexts. Both studies began recruitment in the early 2000’s, thus, the data used in this study was collected between 10-23 years ago. This means that children included in these studies are almost a generation older than children being born today. Socially “normative” parenting practices are thought to change across generations^46,47^, as does understanding of “typical” child behaviours^48^. It is possible that associations between parents and children born in the early 2000 s may look different to those born today. If true, this could have implications for how our findings may translate to interventions or clinical practice in today’s children and parents.

Longitudinal twin research has shown how genetic influences on aggressive and rule-breaking behaviours develop through early childhood into adolescence, before stabilising^10,49,50^. Given the genetic relatedness found here between child aggression and parenting behaviours, it is plausible that the contribution of shared genetics to the parent-child associations may not be static across child development. Therefore, the findings presented regarding the aetiology of intergenerational transmission should be expanded to different timepoints across child development and early adulthood to better understand the complex interaction between parent and child across the lifespan.

In conclusion, across two different samples and study designs, we found evidence for the role of genetics in the relationship between both inconsistent discipline and positive parenting behaviours with child aggression. We also saw child-to-parent effects, whereby aggressive behaviour in childhood evoked higher levels of inconsistent discipline in their mothers. Taken together, these results underscore the importance of using genetically informed samples when studying relationships between parenting behaviours and child outcomes. Research which does not control for the genetic relatedness of individuals runs the risk of misidentifying genetic associations as evidence for behavioural effect, thus blurring the potential for identifying true environmental targets for intervention. However, it is also important to note that the impact of genes on behaviour, or transmission of behaviour, is not static, and is intricately linked with environmental influences which change across the lifetime. Further, this work shows the strength of triangulation, allowing us to describe a pattern of intergenerational association that is not dependent on specific samples or study design. This work is crucial to building a convincing picture of the ways in which parents and children interact, which in turn will hopefully lead to more successful targets for intervention in the development of difficulties within the family, and as the child grows up.

## Methods

Analyses detailed below were pre-registered at 10.17605/OSF.IO/CYQ23. Unregistered sensitivity analyses are flagged in the text. The establishment of MoBa and initial data collection was based on a license from the Norwegian Data Protection Agency and approval from the Regional Committees for Medical and Health Research Ethics. The MoBa cohort is currently regulated by the Norwegian Health Registry Act.

Ethical approval for EGDS data collection was obtained from the three institutional review boards for the universities involved in data collection, Penn State University, Oregon Social Learning Centre, and George Washington University.

Data analysis was conducted on de-identified data at King’s College London (KCL). At KCL, analysis of de-identified secondary data is exempt from the need for further ethical approval by the KCL Research Ethics Committee.

### Samples and measures

No statistical method was used to predetermine sample size.

### Participants

MoBa is a population-based pregnancy cohort study conducted by the Norwegian Institute of Public Health. Participants were recruited from all over Norway between 1999–2008, with a recruitment rate of 41% of all pregnant women contacted during this time. Participants gave informed consent for themselves and their children, with questionnaires administered regularly over the following 20 years. The cohort includes approximately 114,500 children, 95,200 mothers and 75,200 fathers, with a mean maternal age at birth of 27.6 (SD = 4.6)^51^. Mothers had on average 15.23 years of education (SD = 2.27). The mean annual income after tax for this sample at first contact was 744,551 NOK (SD = 45,396 NOK), which is significantly higher than the Norwegian population average, and converts to ~ $74,000^52^. The current study is based on version 12 of the quality-assured data files. The establishment of MoBa and initial data collection was based on a license from the Norwegian Data Protection Agency and approval from the Regional Committees for Medical and Health Research Ethics. The MoBa cohort is currently regulated by the Norwegian Health Registry Act. We extracted extended families from MoBa, which comprised monozygotic (MZ) and dizygotic (DZ) twins, siblings, half-siblings and cousins in both the parent and child generation. Zygosity of twins was determined based on genotyping, questionnaires, and linkage to the Norwegian Twin Registry. In total, raw data were used for 53,532 children born to 42,538 mothers (see Supplementary Table 1 for a full overview of pedigree structures). For the present analyses, we adapted the MCoTS model^27^, for use with the MoBa family structure.

The Early Growth and Development Study (EGDS) is a US prospective study following adopted children, and their adoptive and biological parents (*N* = 561 triads) recruited in two cohorts between 2000–2009, with regular assessments over the following 21 years. Children were adopted within 3 months of birth (median age at placement = 2 days), into non-biologically related families. Adopted children were 57% male. Adoptive mothers were an average of 37.8 years old (standard deviation = 5.50) at the time of the adopted child’s birth, with median educational attainment at the college degree level. Adoptive families had a median annual income of $100,000-$125,000^53^. To be consistent with the MoBa component of this study, we used mother reports of parenting and child outcomes. In sensitivity analyses, we reran analyses using paternal reports to explore to what extent associations between parent and child differed depending on parent gender. In MoBa, we used data from the questionnaire completed when children turned 5 years. For the longitudinal analyses in EGDS, we included data collected at 4.5 years, 6 years, 7 years, and 8 years (see Supplementary Table 9 for sample sizes at each time point). After receiving a complete description of their participation, all parents provided written informed consent for themselves, and adoptive parents consented for their child. Ethical approval was obtained from the three institutional review boards for the universities involved in data collection.

Data analysis was conducted on de-identified data at King’s College London (KCL). At KCL, analysis of de-identified secondary data is exempt from the need for further ethical approval by the KCL Research Ethics Committee.

### Measures

Both samples included measures from the Alabama Parenting Questionnaire (APQ)^11^ and the Child Behaviour Checklist (CBCL)^54^. Specifically, both datasets included the APQ positive involvement and inconsistent discipline subscales, both of which have previously been correlated with child aggression^11^ and the CBCL aggressive behaviours subscale. We focused solely on these dimensions to allow for triangulation across studies. EGDS included the APQ and CBCL subscales in full, and MoBa included a selection of items from each.

Child aggressive behaviours: Both datasets included the aggressive behaviour subscale of the CBCL, which aims to assess problem behaviour in children and asks about violent behaviours such as whether the child “gets in many fights” or shows defiance (“punishment doesn’t change behaviour”). MoBa included 5 items from the aggressive behaviour’s subscale of the CBCL (1.5-5; Cronbach’s α = 0.57), which we used to create a latent factor (described below in more detail). In MoBa, only maternal reports were available for the 5-year questionnaire.

In EGDS, there were two versions depending on the child’s age: both cohorts used the CBCL (1.5-5) aggressive behaviour subscale (19 items) when the child was 4.5 (Cronbach’s α = 0.90), and cohort 1 used this version when the children were 6 years (Cronbach’s α = 0.90). The CBCL (6–18) aggressive behaviour subscale (18 items) at age 6 for cohort 2 (α = 0.90), then for both cohorts at 7 and 8 years (Cronbach’s α = 0.88, 0.89). Both adoptive mother and father reports were available for child behaviour using the CBCL.

Parenting: Inconsistent discipline items cover themes of threatening punishment without following through, such as “You threaten to punish your child and then do not actually punish him/her”. Positive parenting items ask about experiences of positive reinforcement, e.g., “You compliment your child when he/she has done something well”. MoBa included 3 items from the APQ positive parenting subscale (Cronbach’s α = 0.75), and 3 items from the APQ inconsistent discipline subscale (Cronbach’s α = 0.65), via maternal self-report. EGDS included all 6 items for each (Cronbach’s α = 0.62-0.76), reported on by both adoptive mothers and fathers.

Latent factor specification for MoBa analyses: For the analyses using the MoBa study, we specified three latent factors to capture the common variance across the scale items for each construct assessed in parents and children using confirmatory factor analysis in the lavaan() package for R^55^. Measurement error in our traits can impact the ability of the model to correctly identify the direction of causation^29,56^. Therefore, using latent factors allowed us to adjust for item-specific measurement error from the summary score for each measure^29^. We specified one factor per phenotype for inconsistent discipline (3 items loading onto the factor), positive parenting (3 items loading onto the factor) and child aggressive behaviours (5 items loading onto the factor). The factor loading of the first indicator for each phenotype was fixed to 1. The other factor loadings were freely estimated.

Control variables: In MoBa, we regressed maternal age, child year of birth, parity, and child sex-assigned-at-birth out of the parenting dimensions and child aggressive behaviour variables, and the resultant residuals were standardised.

For analyses in the EGDS, we regressed an index of socioeconomic status (SES; created from adoptive parents’ education level and income when the child was 7), adoption openness (a measure of contact between birth and adoptive families), child sex-assigned-at-birth and maternal age from the parenting and child outcome variables. The resultant residuals were standardised and used in the structural equation models.

### Analysis

All analyses were conducted using R version 4.3.1 (2023-06-16)^57^. The extended-family design analyses were run in *OpenMx* version 2.21.8^58^, and the longitudinal adoption models were run using *lavaan* version 0.6-15^55^.

**Fig. 5:**
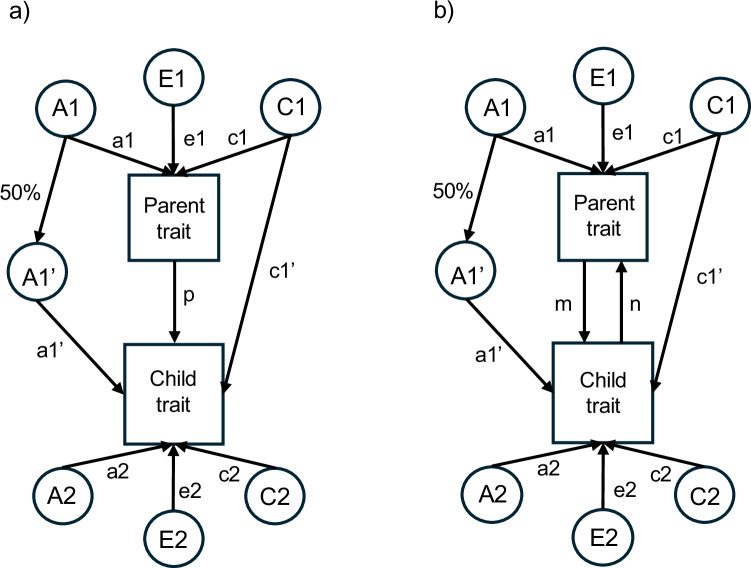
Partial paths diagram for the Multiple-children-of-twins-and-siblings (MCoTS) structural equation models^27,59^. **a** MCoTS model to decomposes intergenerational associations between parenting and child aggressive behaviours into genetic and environmental components. Squares denote observed variables and circles denote latent variables. Note: A1 = additive genetic effects on parental phenotype; C1 = shared-environmental effects on parental phenotype; E1 = nonshared environmental effects on parental phenotype; A1′ = genetic effects common to parental phenotype and offspring phenotype; C1′ = extended family effects whereby the shared environment of the parents influences offspring phenotype; A2 = additive genetic effects specific to offspring phenotype; C2 = shared-environmental effects on offspring phenotype (not estimable using cousin data); E2 = nonshared environmental effects on offspring phenotype; *p* = remaining phenotypic path after controlling for shared genetic and environmental influences. **b** MCoTS model adapted to assess direction-of-causation (MCoTS-DoC). Note: *m *= phenotypic effect of parent on offspring. *n *= phenotypic effect of offspring on parent. NB the pathway between A1 and A1′ is fixed to 0.50 because parents and children share 50% of their genome.

Extended-family design in MoBa: The aim of this design was to estimate the direction of effects between parenting and childhood aggression while accounting for genetic confounding by using cross-sectional extended family data drawn from a large birth cohort. We employed the MCoTS model, which decomposes intergenerational associations between parenting and child aggressive behaviours into genetic and environmental components (Supplementary Fig. 1). We ran a full model (including additive genetic, shared environmental, and non-shared environmental influences on parent trait, child trait, and their covariance), before dropping non-significant paths to find the most parsimonious model that adequately fitted the data. The MCoTS model can account for passive rGE via the a1’ pathway (Fig. 5a), which captures genetic effects common across parent and child phenotypes. The p path (Fig. 5a) indexes the residual parent-child association after controlling for passive rGE.

The MCoTS model was further adapted to assess direction-of-causation (MCoTS-DoC; Fig. 5b)^28,59^. Direction-of-causation twin models can distinguish the likely direction of effects between two traits of differing aetiology^29^. Where two traits have distinct aetiologies (e.g., if one is more heritable than the other), and one trait causes the other, the aetiology of the covariance will reveal which trait causes which. We applied MCoTS-DoC models, where MCoTS models revealed that parent-child associations remained after controlling for shared genetic aetiology. We tested a model which included both the parent-to-child path and child-to-parent path, before dropping one or the other, to identify the best-fitting model.

Sensitivity analyses in MoBa: These analyses were suggested by reviewers, and so were not pre-registered. In MoBa, we ran sex-stratified sensitivity analyses, where we selected mothers with only male or female children to build the family structures around. In this way, we could run the MCoTS models for mothers reporting on parenting their male children only, and then separately for mothers reporting on parenting their female children. As these were not nested models, we could not compare model fit, however we tested sex differences by comparing confidence intervals for each variance component across the two sexes.

Longitudinal analyses in the EGDS adoption sample: Here, we used longitudinal designs to estimate the direction of effects between parenting and child aggression while accounting for genetic confounding using adoptive families. We employed two longitudinal models which employ the use of random intercepts to separate out stability in parenting and childhood aggression from timepoint-specific variation. In the first design, parent-child effects were modelled as occurring within timepoint and in the second, they were modelled across time.

**Fig. 6:**
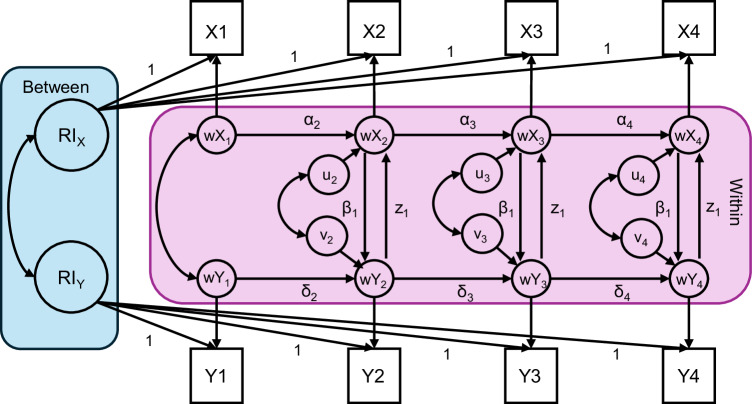
Testing parent-child effects within time with a bivariate autoregressive fixed effect model^60,61^. Note: Squares denote observed variables and circles denote latent variables. One-headed arrows denote regression paths. X_t _= child aggression and Y_t _= parenting behaviour (inconsistent discipline or positive parenting) at timepoint t. RI = Random intercept/time invariant variance for X and Y, i.e., the between-cluster level shown in the blue box. The within-cluster level is shown in the pink box, where wXt and xYt denote time variant variance, and a_t_ and d_t_ denote autoregressive paths where each time point is regressed on the previous time point. β_1_ and Z_1_ denote the reciprocal effect of parent on child and vice versa.

Analysis 1: Parent-child effects within time. For many traits (parenting and aggressive behaviours included), it is possible that causal processes unfold over short time periods. For example, effects may be detectable over periods of hours, days or months, but may not persist over several years. For this reason, we applied a bivariate autoregressive fixed effect model^60,61^ (see Fig. 6) to EGDS data, which allowed for a test of the likely direction of effects between mother and child without the requirement that prediction persists across long time periods (as required in a cross-lagged panel model). Parent-to-child path estimates were set to be equal across timepoints, as were the child-to-parent paths. This model could then estimate cross-sectional bidirectional effects. We reasoned that this approach would be best suited to our stated aim of triangulating results with the cross-sectional MCoTS-DoC design.

**Fig. 7:**
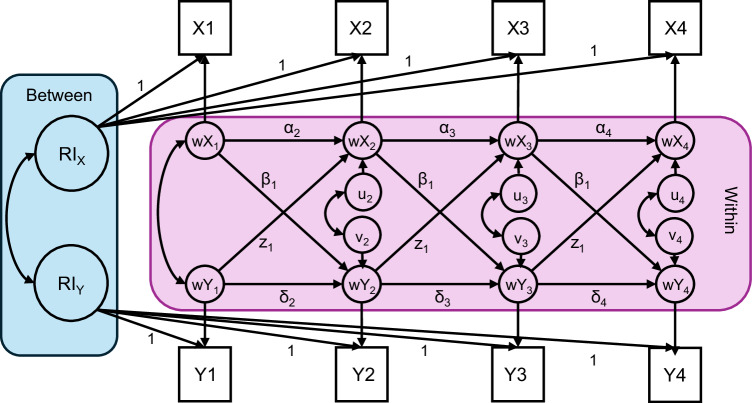
Testing parent-child effects across time with a random-intercept cross-lagged panel model effect model^63^. Note: Squares denote observed variables and circles denote latent variables. One-headed arrows denote regression paths. X_t _= child aggression and Y_t _= parenting behaviour (inconsistent discipline or positive parenting) at timepoint t. RI = Random intercept/time invariant variance for X and Y, i.e., the between-cluster level shown in the blue box. The within-cluster level is shown in the pink box, where wXt and xYt = time variant variance, and a_t_ and d_t _= autoregressive paths, where each time point is regressed on the previous time point. β_1_ and Z_1 _= the cross-lags of parent on child and vice versa. u_t_ and v_t _= remaining covariance between variables.

Analysis 2: Parent-child effects across time. Cross-lagged panel models are frequently applied to longitudinal observational data as a method for determining the direction of effects between associated variables. To situate our findings within that literature, we used a (random-intercept) cross-lagged panel model (RI-CLPM)^62^ as a point of comparison to the bivariate autoregressive fixed effect model (Fig. 7). Cross-lagged panel models allow the estimation of prospective effects of one variable on another; thus, we can infer the direction-of-effects (parent-to-child vs child-to-parent), and whether these effects persist over these time lags. The random intercept captures time-invariant stability in parenting and in aggression. Therefore, residual effects are time-specific variations. The model asks whether a parent’s time-specific variation around their mean parenting (the random intercept) influences variation around the child’s mean aggression (random intercept), and vice versa. Again, parent-to-child path estimates were set to be equal across timepoints, as were the child-to-parent paths.

Sensitivity analyses in EGDS: In EGDS, we had access to paternal reports of parenting and child outcomes. We made use of the second reporter to conduct sensitivity analyses using (i) paternal reports of parenting behaviours and child aggression; (ii) paternal reports of child aggression in mother-child dyads, (iii) maternal reports of child aggression father-child dyads. Analyses using paternal reports for both parent and child variables were preregistered. Analyses (ii) and (iii) were run following the suggestion of an anonymous reviewer, and so were not preregistered.

To test for child sex differences, we incorporated child sex as a categorical grouping variable in *lavaan*. Multi-group models were then estimated by specifying the sex variable in the group argument. We then ran one model with parameters allowed to be freely estimated across the two sexes, and then a second where the paths of interest (parent-to-child and child-to-parent) were constrained to be equal across sexes. Again, these analyses were suggested during the review process and so were not preregistered. As the models were nested, we could compare model fit, where a finding of a non-significant difference in fit between the unconstrained and constrained models would suggest no sex differences in paths.

### Reporting summary

Further information on research design is available in the Nature Portfolio Reporting Summary linked to this article.

## Supplementary information

Supplementary Information

Reporting Summary

Transparent Peer Review file

## Data Availability

MoBa data is not publicly available because the consent given by the participants does not allow for data storage on an individual level in repositories or journals. MoBa data can be accessed by application to the Regional Committee for Medical and Health Research Ethics in Norway and MoBa (https://www.fhi.no/en/ch/studies/moba/for-forskere-artikler/research-and-data-access/). Fully deidentified EGDS data has been uploaded to OSF and is available at 10.17605/OSF.IO/DVXNC.

## Code availability

Code used in the analysis of data is available at 10.17605/OSF.IO/DVXNC
