# Supplementary material for: Triangulating evidence for genetic and environmental components of associations between parental behaviours and aggressive behaviour in children
Source: Nat Commun. 2026 Jul 6;17:8340. doi: 10.1038/s41467-026-75095-5 (PMC13473577; doi:10.1038/s41467-026-75095-5)
Supplement: Supplementary file 1 [file 41467_2026_75095_MOESM1_ESM.pdf]

Supplementary Information
Methods

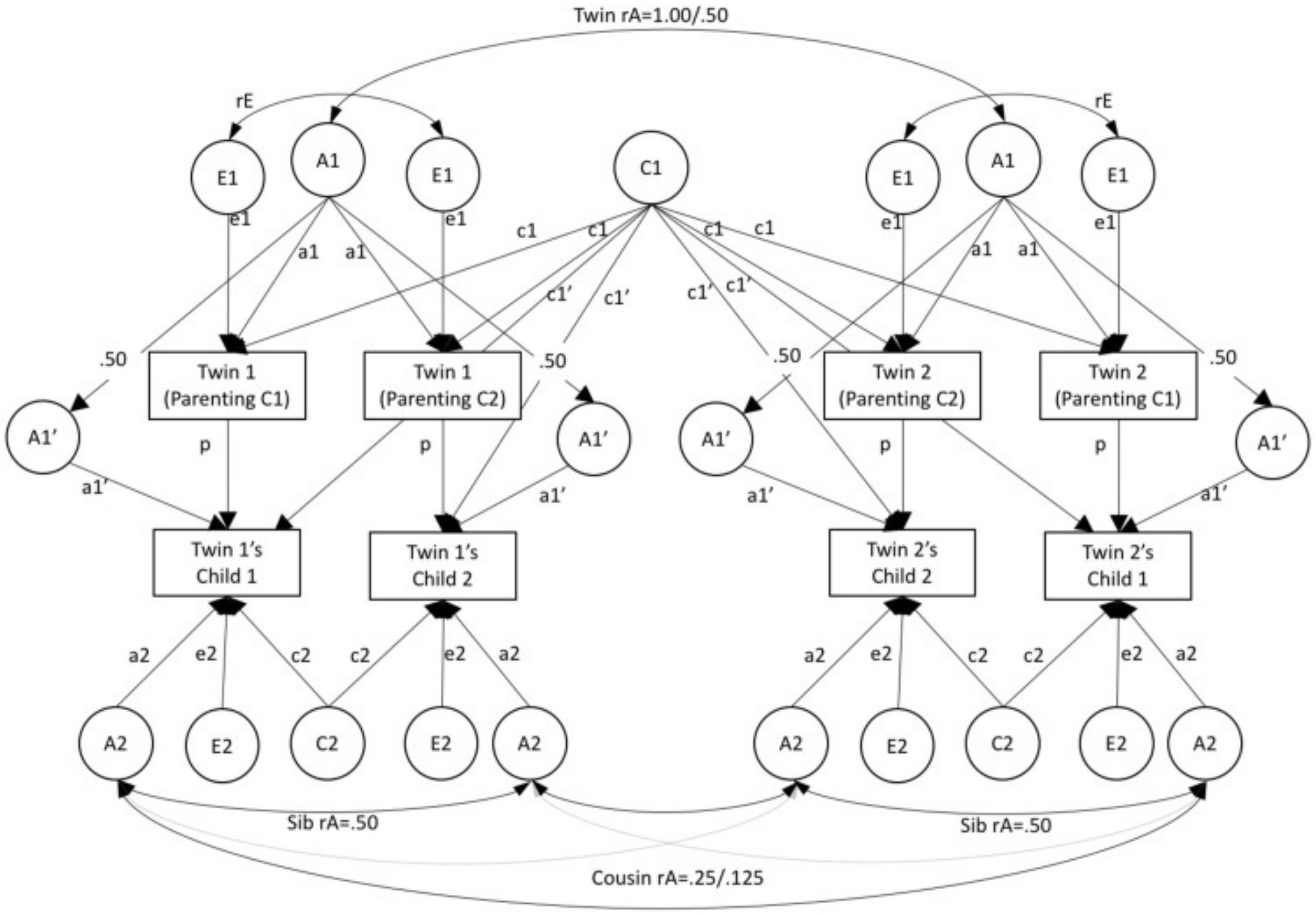

Supplementary Figure 1: Full path diagram for the Multiple-children-of-twins/siblings (MCoTS) structural equation model. Note: A1 = additive genetic effects on parental phenotype; C1 = shared-environmental effects on parental phenotype; E1 = nonshared environmental effects on parental phenotype; A1′ = genetic effects common to parental phenotype and offspring phenotype; C1′ = extended family effects whereby the shared environment of the parents influences offspring phenotype; A2 = additive genetic effects specific to offspring phenotype; C2 = shared-environmental effects on offspring phenotype (not estimable using cousin data); E2 = nonshared environmental effects on offspring phenotype; m = phenotypic effect of parent on offspring. n = phenotypic effect of offspring on parent. Within-parent correlation between E1 for parenting of child 1 and 2 (rE) allows parenting of each child to differ (when necessary, this should be allowed to vary according to offspring zygosity). NB the pathway between A1 and A1′ is fixed to 0.50 because parents and children share 50% of their genome.

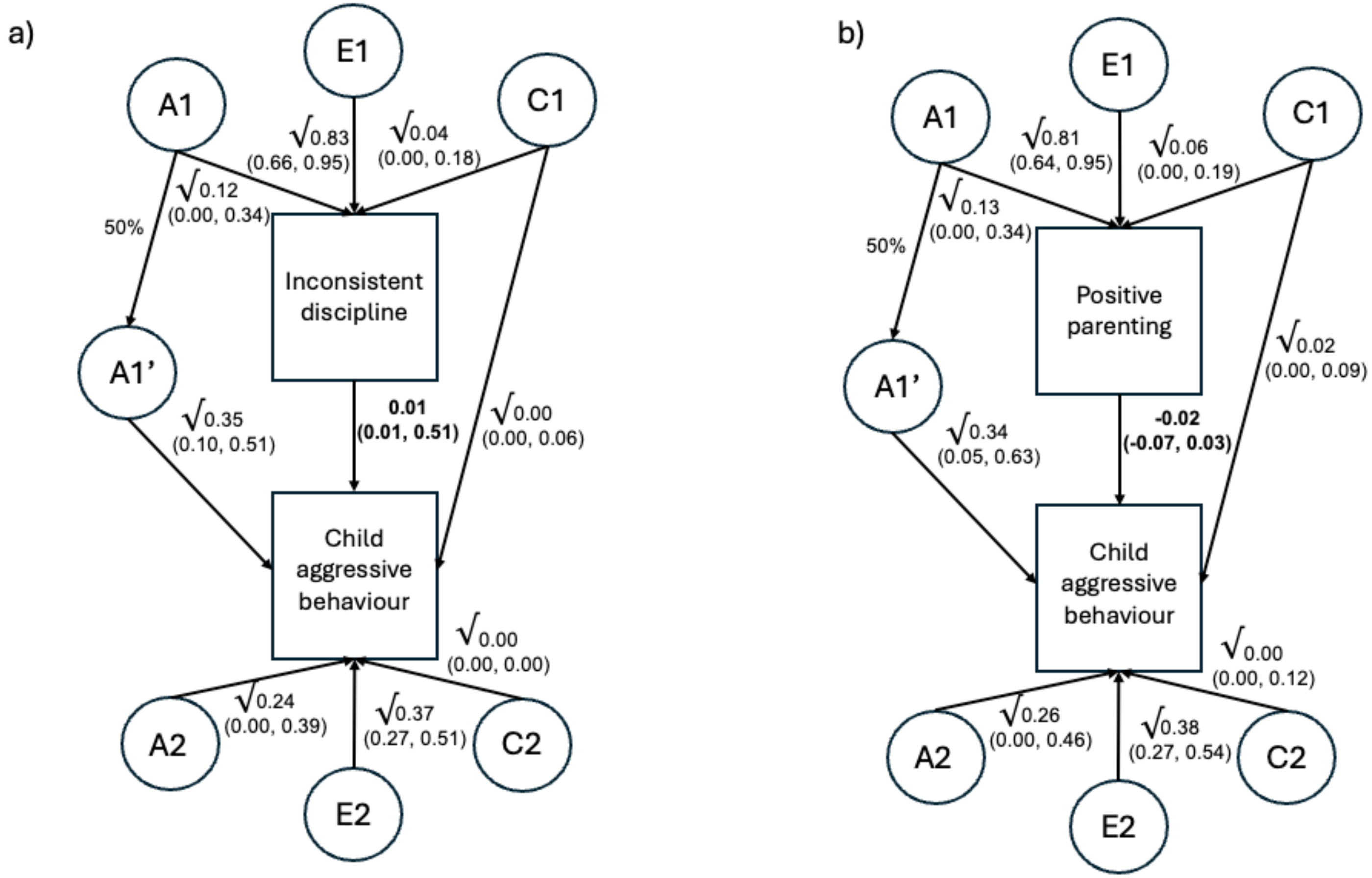

Supplementary Figure 2: Results from the Multiple-Children-of-Twins/Siblings structural equation model for a) inconsistent discipline & b) positive parenting and child aggressive behaviours. Figures represent standardised path estimates (95% confidence intervals) from the full model decomposing the association between mothers' inconsistent discipline and child aggressive behaviour (*r* = 0.22), and mothers' positive parenting and child aggressive behaviour (*r* = -0.09). Squares denote observed variables and circles denote latent variables. Variance components are displayed in non-bold text. The residual intergenerational association is displayed as a standardised path beta coefficient in **bold text**.

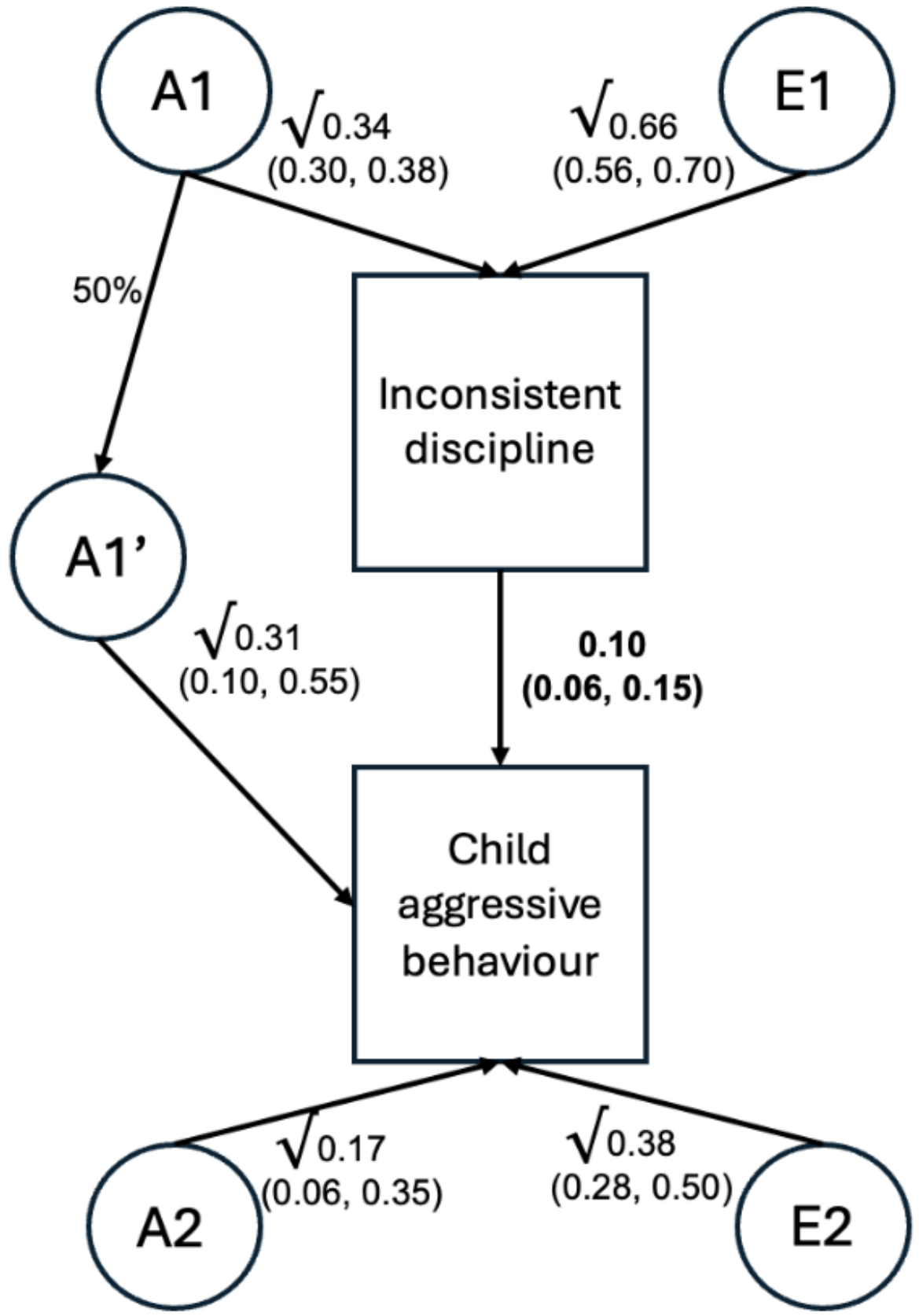

Supplementary Figure 3: Results from the Multiple-Children-of-Twins/Siblings structural equation model. Figure represents standardised path estimates (95% confidence intervals) from the best fitting model decomposing the association between mothers' inconsistent discipline and child aggressive behaviour ($r = 0.22$). Squares denote observed variables and circles denote latent variables. A1, additive genetic effects on mother behaviour; A2, additive genetic effects specific to child behaviour; A1′, additive genetic effects common to mother and child behaviours (path from A1 to A1′ is fixed to 0.50 because offspring inherit 50% of their mother's genes); E1/E2, unique environment effects on mother/child behaviours. Variance components are displayed in non-bold text. The residual intergenerational association is displayed as a standardised path beta coefficient in bold text.

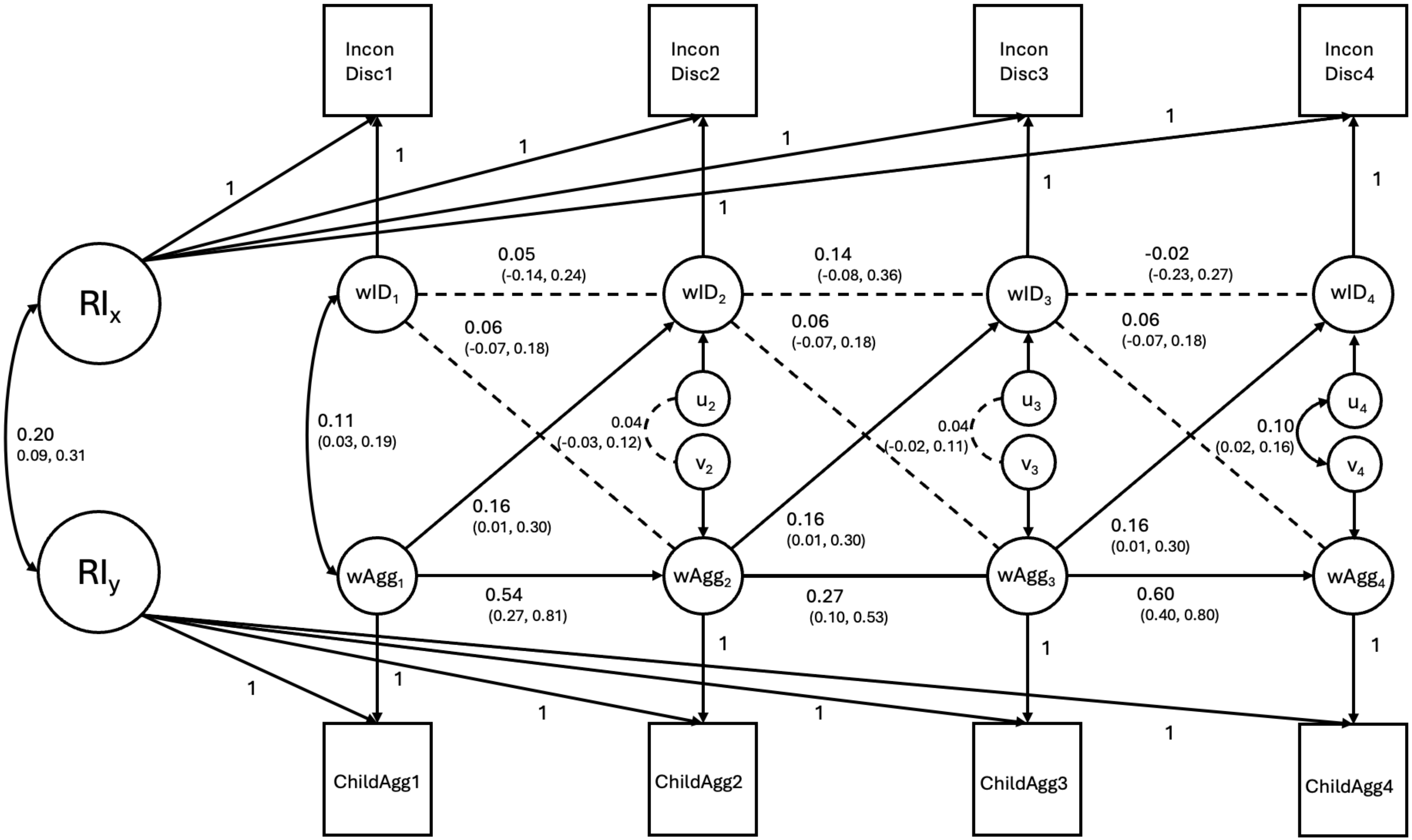

Supplementary Figure 4: Random intercept cross-lagged panel models for inconsistent discipline and aggression. Figure represents standardised path estimates (95% confidence intervals) from the best fitting model for the testing the effects of mothers' inconsistent discipline (ID) and child aggressive behaviour (Agg) across time. Squares denote observed variables and circles denote latent variables. Note: RI = Random intercept. Significant paths are in black, non-significant paths are shown by dotted lines.

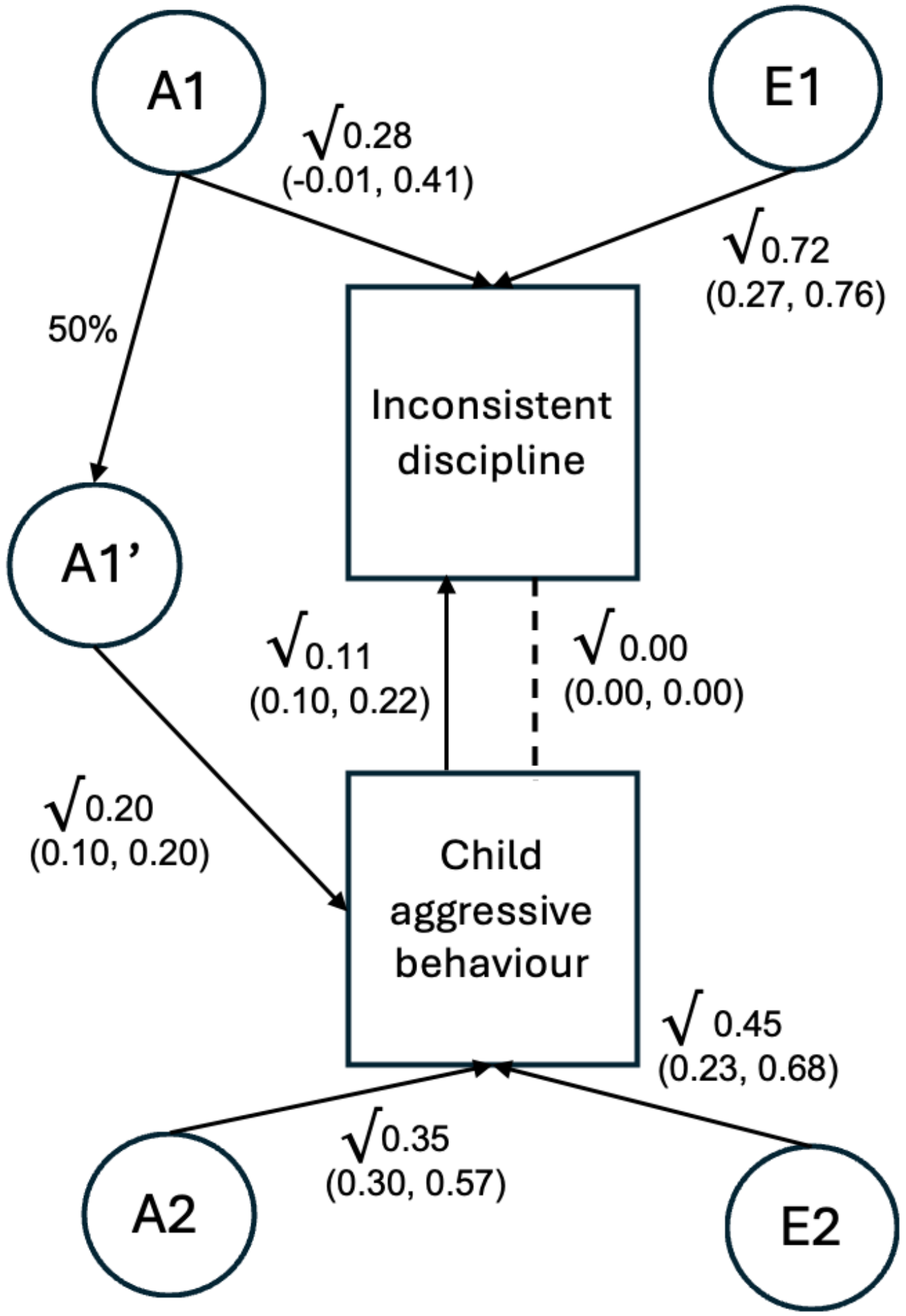

Supplementary Figure 5: Results from MCoTS-DoC bidirectional model, for inconsistent discipline and child aggressive behaviours. Figure represents standardised path estimates (95% confidence intervals) from the the bidirectional model of the association between mothers' inconsistent discipline and child aggressive behaviour (*r* = 0.22). This model estimates both the parent-to-child and child-to-parent simultaneously. Squares denote observed variables and circles denote latent variables. Variance components are displayed in non-bold text. Non-significant parent-to-child path is indicated by thre dotted line.

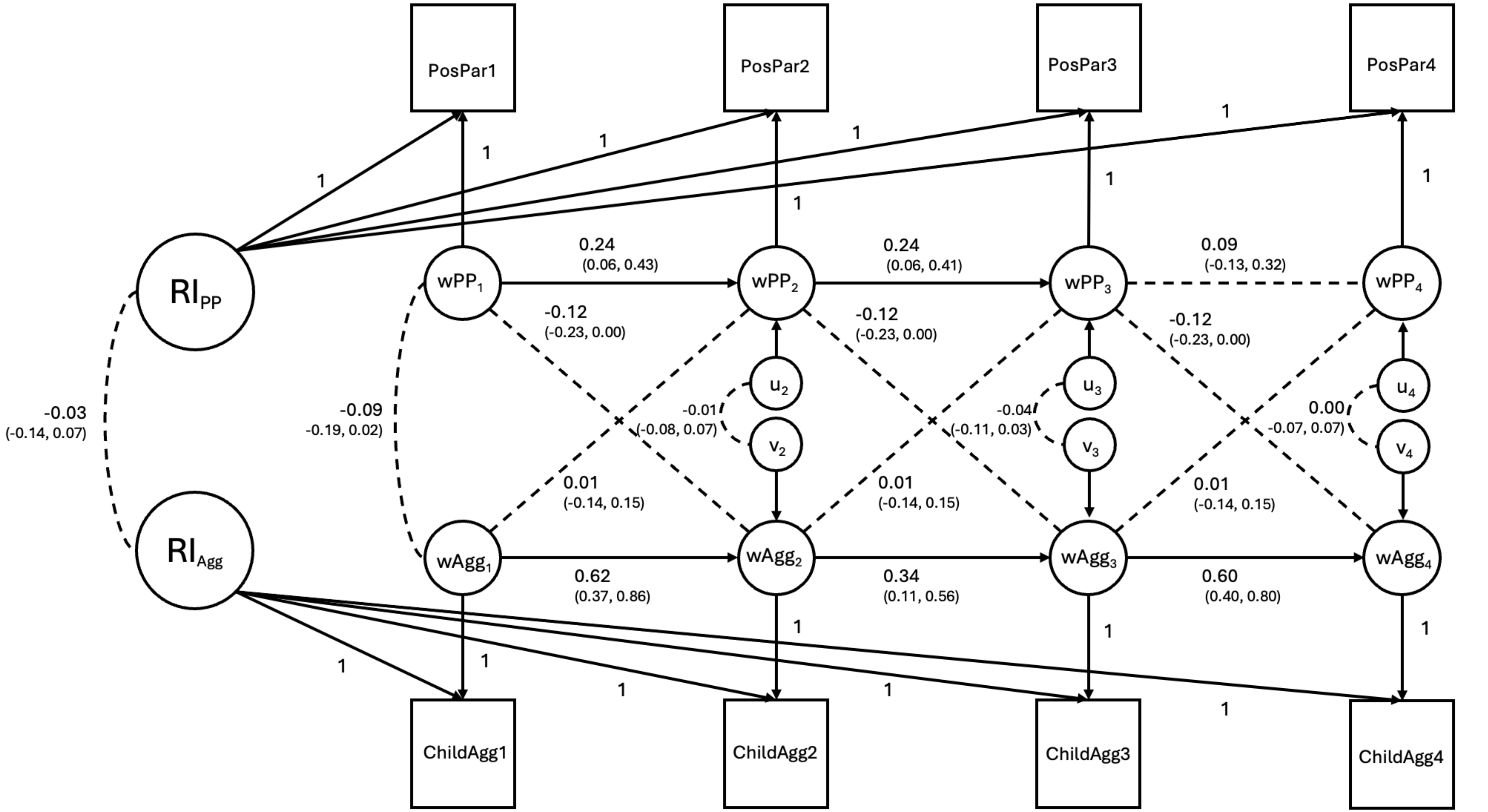

Supplementary Figure 6: Random intercept cross-lagged panel models for positive parenting and aggression. Figure represents standardised path estimates (95% confidence intervals) from the best fitting model for the testing the effects of mothers' positive parenting (PP) and child aggressive behaviour (Agg) across time. Squares denote observed variables and circles denote latent variables. Note: RI = Random intercept. Significant paths are in black, non-significant paths are indicated by dotted lines.

Supplementary Table 1: Overview of pedigree structures and sample sizes used for Multiple-Children-of-Twins/Siblings model analyses.

**Extended families (*N* families= 24,250)[a]**	***n***
**N stratified by the parent pairs used to identify extended families**	
Identical twin pair (100% genetic relatedness)	75
Full-sibling or fraternal twin pair (50% genetic relatedness)	14,942
Maternal/paternal half-sibling pair (25% genetic relatedness)	917
Cousin pair (12.5% genetic relatedness)	8,312
**N stratified by mothers' relatedness in each extended family [b]**	
Identical twin pair (100% genetic relatedness)	52
Full-sibling or fraternal twin pair (50% genetic relatedness)	4,965
Maternal/paternal half-sibling pair (25% genetic relatedness)	362
First cousin pair (12.5% genetic relatedness)	3,541
Unrelated sisters-/cousins-in-law pair (0% genetic relatedness)	15,330
**Number of offspring pairs linked to each mother**	
Full-sibling pair (50% genetic relatedness)	6005
Maternal half-sibling pair (25% genetic relatedness)	59
Unpaired (single) offspring	31,553
**Unpaired nuclear families (N families= 4,921) [c]**	
**Number of offspring pairs linked to each mother**	
Identical twin pair (100% genetic relatedness)	109
Full-sibling or fraternal twin pair (50% genetic relatedness)	4,796
Maternal half-sibling pair (25% genetic relatedness)	16

Note: Numbers represent families who had data for at least one individual in our biometric analyses.
[a]Extended families were identified via adult twin, sibling or cousin pairs enrolled in the MoBa cohort as parents. These included mother-mother, mother-father, or father-father pairs. [b]Phenotypic data were derived from mothers in each extended family. Mothers were genetically unrelated if their extended family was identified using a mother-father or father-father pair (i.e., mothers in these families were sister-in-law or cousin-in-law pairs). [c]Mothers who could not be paired into an extended family (i.e., because they did not have a twin, sibling, cousin, sister-in-law, or cousin-in-law in the dataset) were modelled independently in nuclear family units if they had more than one offspring. Mothers who had twin offspring were also modelled independently in nuclear family units.

Supplementary Table 2: Descriptive Statistics for the MoBa sample

	Mean	Standard Deviation	Cronbach's alpha
Aggression (CBCL) age 5	1.29	0.30	0.57
Positive Parenting (APQ) age 5	4.52	0.44	0.75
Inconsistent discipline (APQ) age 5	2.16	0.62	0.65

Note: APQ = Alabama Parenting Questionnaire; CBCL = Child Behaviour Checklist

Supplementary Table 3: Confirmatory Factor Analysis parameter estimates for the latent factors using MoBa data

Phenotype Factor	Item	Factor Loading Estimate	Standard Error
Positive Parenting	Let your child know they are doing a good job	1.000	-
	Compliment your child	1.245	0.016
	Praise your child	1.433	0.018
Inconsistent Discipline	Threaten to punish your child and then do not actually punish them	1.000	-
	Child talks you out of being punished	0.900	0.016
	Let your child out of a punishment early	0.983	0.018
Aggressive Behaviour	Defiant	1.000	-
	Doesn't seem to feel guilty	0.766	0.011
	Gets in many fights	1.064	0.017
	Hits others	0.413	0.012
	Punishment doesn't change behaviour	0.774	0.014

Supplementary Table 4: Confirmatory Factor Analysis fit statistics for the creation of latent factors for MoBa data

Fit indices	Inconsistent Parenting	Positive Parenting	Child Aggressive Behaviours
CFI	1.00	1.00	0.95
TLI	1.00	1.00	0.90
RMSEA	0.00	0.00	0.07

Note: RMSEA = Root-Mean-Square Error of Approximation; CFI = Comparative Fit Index; TLI = Tucker-Lewis Index. Model fit indices were evaluated such that acceptable fit was determined when CFI > 0.90, the TLI > 0.90 and RMSEA < 0.08 (Hu & Bentler, 1999).

Supplementary Table 5. Correlations between phenotype latent factors in the MoBa sample

	Twin Inconsistent discipline (APQ)	Twin Positive parenting (APQ)
Twin inconsistent discipline (APQ)		-0.114
Offspring aggressive/rule breaking behaviour (CBCL)	0.225	-0.086

Note: Pearson correlation coefficient. All remain significant to at least the p<.05 level after Bonferroni correction for multiple testing. APQ = Alabama Parenting Questionnaire; CBCL = Child Behaviour Checklist

Supplementary Table 6. Descriptive Statistics for the EGDS sample

	N	Mean	Standard Deviation	Cronbach's alpha
Child aggression (CBCL) age 4.5	417	10.39	6.15	0.90
Child aggression (CBCL) age 6	266	8.08	5.49	0.88
Child aggression (CBCL) age 7	397	5.58	4.91	0.88
Child aggression (CBCL) age 8	239	5.43	4.99	0.89
Inconsistent discipline (APQ) age 4.5	436	2.00	0.48	0.71
Inconsistent discipline (APQ) age 6	445	2.01	0.47	0.73
Inconsistent discipline (APQ) age 7	446	2.05	0.49	0.65
Inconsistent discipline (APQ) age 8	239	2.04	0.47	0.62
Positive parenting (APQ) age 4.5	439	4.40	0.39	0.73
Positive parenting (APQ) age 6	446	4.39	0.41	0.76
Positive parenting (APQ) age 7	447	4.34	0.40	0.74
Positive parenting (APQ) age 8	239	4.36	0.40	0.68

Note: APQ = Alabama Parenting Questionnaire; CBCL = Child Behaviour Checklist

Supplementary Table 7. Pairwise correlations between variables in the EGDS sample

		1	2	3	4	5	6	7	8	9	10	11
Child	1. Aggression (CBCL) age 4.5											
Child	2. Aggression (CBCL) age 6	**0.76**										
Child	3. Aggression (CBCL) age 7	**0.65**	**0.66**									
Child	4. Aggression (CBCL) age 8	**0.61**	**0.63**	**0.77**								
Parent	5. Inconsistent discipline (APQ) age 4.5	**0.27**	**0.30**	0.15	**0.19**							
Parent	6. Inconsistent discipline (APQ) age 6	**0.25**	**0.31**	**0.29**	**0.21**	**0.66**						
Parent	7. Inconsistent discipline (APQ) age 7	**0.23**	**0.30**	**0.25**	**0.15**	**0.63**	**0.71**					
Parent	8. Inconsistent discipline (APQ) age 8	**0.34**	**0.27**	**0.23**	**0.30**	**0.53**	**0.63**	**0.57**				
Parent	9. Positive parenting (APQ) age 4.5	**-0.18**	-0.12	-0.12	-0.13	-0.13	-0.07	-0.08	-0.04			
Parent	10. Positive parenting (APQ) age 6	-0.11	-0.03	-0.13	-0.13	-0.08	-0.11	-0.11	-0.07	**0.65**		
Parent	11. Positive parenting (APQ) age 7	-0.08	0.01	-0.12	-0.12	**-0.20**	-0.14	**-0.15**	-0.14	**0.61**	**0.67**	
Parent	12. Positive parenting (APQ) age 8	-0.03	0.08	0.03	0.00	**-0.14**	-0.09	-0.07	-0.13	**0.53**	**0.53**	**0.58**

Note: Pearson correlation coefficient. ***bold*** *indicates significant to at least the p<.05 level after Bonferroni correction for multiple testing. Cross-time correlations within construct are highlighted in dark grey. Correlations between parent and child measures are highlighted in light grey. APQ = Alabama Parenting Questionnaire; CBCL = Child Behaviour Checklist*

Supplementary Table 8. Observed correlations between parenting dimensions and child aggressive behaviours, decomposed into genetic and non-genetic components via the MCoTS model

	Correlation with child aggressive behaviours	Genetic component	Non-genetic component
Inconsistent Discipline	0.22	47%	53%
Positive Parenting	-0.10	100%	0%

Note: The genetic component is computed from the additive genetic (A) paths, and the non-genetic component is calculated using the common environmental (C) and unique environmental (E) paths. In this model, the non-genetic component comes from E only, as the C paths were dropped.

Supplementary Table 9. Model Fit statistics for MCoTS and MCoTS-DoC models for parenting behaviours and child aggression

Multiple Children of Twins/Siblings model

Inconsistent discipline	Estimated parameters	-2LL	AIC	df	Diff LL	p-value
Full model	12	106,093.70	106,117.70	38,172		
**No shared environment (C) paths**	**9**	**106,094.00**	**106,112.00**	**38,175**	**0.28**	**0.964**
Phenotypic Transmission only (No genetic path)	8	106,114.10	106,130.10	38,176	20.43	0.000
Genetic Transmission only (No phenotypic path)	8	106,113.00	106,129.50	38,176	19.74	0.001
Positive parenting	Estimated parameters	-2LL	AIC	df	Diff LL	p
Full model	12	107,145.40	107,169.40	38,187		
No shared environment (C) paths	9	107,148.40	107,166.40	38,190	3.009	0.390
Phenotypic Transmission (No genetic path)	8	107,157.20	107,173.20	38,191	16.641	0.019
**Genetic Transmission (No phenotypic path)**	**8**	**107,148.40**	**107,164.40**	**38,200**	**3.038**	**0.552**

Multiple Children of Twins/Siblings – Direction of Causation model

Inconsistent discipline	Estimated parameters	-2LL	AIC	DF	Diff LL	p-value
Bi-directional (both Parent-child transmission and Child-parent transmission)	11	106,027.40	106,049.40	38,173		
Parent-child transmission (no Child-parent transmission)	10	106,079.40	106,099.40	38,174	51.95	0.000
**Child-parent transmission (no Parent-child transmission)**	**10**	**105,955.50**	**105,975.70**	**38,174**	**-71.91**	**1.000**

*Note: Model comparison tested using the likelihood ratio test. -2LL = minus 2 Log likelihood; AIC = Akaike information criterion; df = degrees of freedom; Diff LL = difference in -2LL. Rows in **bold** are the final model, selected as the most parsimonious model*

Supplementary Table 10. Structural equation model ACE components from MCoTS models and MCoTS-DoC models for parenting behaviours and child aggression by child sex-assigned-at-birth

	**A1**	**C1**	**E1**	**A1'**	**C1'**	**A2**	**C2**	**E2**	**p**
Inconsistent Discipline (boys)	0.04 (0.00, 0.46)	0.26 (0.00, 0.34)	0.79 (0.52, 0.96)	0.50 (0.11, 0.76)	0.01 (0.00, 0.09)	0.13 (0.00, 0.40)	0.00 (0.00, 0.12)	0.32 (0.19, 0.49)	0.11 (0.04, 0.23)
Inconsistent Discipline (girls)	0.01 (0.00, 0.51)	0.07 (0.00, 0.28)	0.93 (0.48, 1.00)	0.43 (0.00, 0.70)	0.04 (0.00, 0.16)	0.06 (0.00, 0.41)	0.00 (0.00, 0.14)	0.41 (0.25, 0.71)	0.14 (0.02, 0.26)
Positive Parenting (boys)	0.15 (0.00, 0.05)	0.02 (0.00, 0.49)	0.83 (0.50, 1.00)	0.44 (0.06, 0.77)	0.01 (0.00, 0.13)	0.22 (0.00, 0.50)	0.00 (0.00, 0.13)	0.33 (0.21, 0.53)	0.04 (-0.06, 0.14)
Positive Parenting (girls)	0.00 (0.00, 0.40)	0.11 (0.00, 0.26)	0.89 (0.72, 1.00)	0.45 (0.00, 0.72)	0.05 (0.00, 0.17)	0.04 (0.00, 0.45)	0.02 (0.00, 0.16)	0.43 (0.26, 0.70)	-0.03 (-0.13, 0.07)

Note: Standardised path estimates (95% confidence intervals). A1 = additive genetic effects on parental phenotype; E1 = nonshared environmental effects on parental phenotype; A1′ = genetic effects common to parental phenotype and offspring phenotype; A2 = additive genetic effects specific to offspring phenotype; E2 = nonshared environmental effects on offspring phenotype; p = remaining behavioural association; m = phenotypic effect of parent on offspring. n = phenotypic effect of offspring on parent. C1, C1' and C2 paths (shared-environmental effects on parent & offspring phenotypes were non-significant in the full model and so were dropped, thus have not been reported here.

Supplementary Table 11. Model Fit statistics for MCoTS and MCoTS-DoC models for parenting behaviours and child aggression by child sex-assigned-at-birth

	**-2LL**	**AIC**	**df**
Inconsistent discipline (boys)	50,224.72	50,248.72	17,699
Inconsistent discipline (girls)	45,446.04	45,470.04	16,668
Positive parenting (boys)	50,794.74	50,818.74	17,721
Positive parenting (girls)	45,899.26	45,923.26	16,696

Note: Each model had 12 estimated parameters. 2LL = minus 2 Log likelihood; AIC = Akaike information criterion; DF = degrees of freedom

Supplementary Table 12: Main results from sensitivity analyses in the EGDS adoption sample.

Sensitivity analyses			Path estimates			
			Parent-to-child		Child-to-parent	
**Parenting dimension**	**Parenting reporter**	**Child aggression reporter**	**Cross lags**	**Within-timepoint**	**Cross lags**	**Within-timepoint**
Inconsistent discipline	Father reported	Father reported	-0.08 (-0.20, 0.05)	**0.35 (0.11, 0.59)**	0.06 (-0.10, 0.21)	**1.70 (0.73, 2.68)**
	Father reported	Mother reported	-0.04 (-0.16, 0.08)	0.16 (-0.61, 0.29)	0.03 (-0.12, 0.18)	0.05 (-0.25, 0.34)
	Mother reported	Father reported	0.02 (-0.12, 0.15)	0.61 (-0.49, 1.70)	0.04 (-0.10, 0.18)	0.33 (-0.36, 1.03)
	Mother reported (paths constrained as equal across sex)	Mother reported (paths constrained as equal across sex)	0.20 (-0.05, 0.45)	2.27 (-0.12, 4.66)	**0.10 (0.03, 0.16)**	**0.17 (0.03, 0.31)**
Positive parenting	Father reported	Father reported	-0.04 (-0.18, 0.09)	-0.25 (-0.94, 0.44)	0.02 (-0.13, 0.17)	0.44 (-0.25, 1.13)
	Father reported	Mother reported	0.13 (-0.01, 0.23)	0.25 (-0.22, 0.72)	0.15 (-0.002, 0.30)	0.11 (-0.24, 0.47)
	Mother reported	Father reported	-0.09 (-0.21, 0.04)	-0.60 (-1.44, 0.24)	-0.02 (-0.16, 0.11)	1.93 (-4.08, 7.95)
	Mother reported (paths constrained as equal across sex)	Mother reported (paths constrained as equal across sex)	0.01 (-0.14, 0.15)	-0.70 (-1.57, 0.17)	0.08 (-0.17, 0.32)	0.04 (-0.26, 0.34)

Note: Standardised path estimates (95% confidence intervals). ***Bold*** *indicates significant to at least the p<.05 level.*

Supplementary Table 13: Fit statistics from sensitivity analyses in the EGDS adoption sample – testing child sex differences.

Parenting dimension	Sex-specific model	Df	AIC	BIC	X^2	Diff X^2	p-value
Inconsistent discipline (cross lagged model)	Mother reported (paths unconstrained across sex)	26	3469.9	3708.0	36.29	-	-
	Mother reported (paths constrained to be equal across sex)	28	3470.8	3701.3	41.12	4.94	0.085
Inconsistent discipline (within time model)	Mother reported (paths unconstrained across sex)	26	4194.8	4432.9	64.31	-	-
	Mother reported (paths constrained to be equal across sex)	28	4191.4	4421.8	64.91	0.33	0.848
Positive parenting (cross lagged model)	Mother reported (paths unconstrained across sex)	26	5427.7	5665.8	29.58	-	-
	Mother reported (paths constrained to be equal across sex)	28	5428.0	5658.4	33.91	4.33	0.115
Positive parenting (within time model)	Mother reported (paths unconstrained across sex)	26	5425.9	5664.0	27.75	-	-
	Mother reported (paths constrained to be equal across sex)	28	5426.6	5657.1	32.53	4.78	0.092

Note: AIC = Akaike information criterion; BIC = Bayesian information criterion; DF = degrees of freedom; χ^2 = Chi-squared difference test statistic (one-tailed) and significance testing.

Supplementary Table 14: Fit statistics from sensitivity analyses in the EGDS adoption sample – maternal and paternal reports.

Sensitivity analyses			Fit statistics					
			CFI		TLI		RMSEA	
Parenting dimension	Parenting reporter	Child aggression reporter	Cross lags	Within-timepoint	Cross lags	Within-timepoint	Cross lags	Within-timepoint
Inconsistent discipline	Father reported	Father reported	0.996	0.964	0.991	0.923	0.029	0.075
	Father reported	Mother reported	1.000	1.000	1.004	1.004	0.000	0.000
	Mother reported	Father reported	1.000	1.000	1.007	1.010	0.000	0.000
Positive parenting	Father reported	Father reported	0.996	0.995	0.990	0.989	0.029	0.031
	Father reported	Mother reported	0.997	1.000	0.993	1.004	0.027	0.000
	Mother reported	Father reported	0.997	0.998	0.993	0.995	0.025	0.021

Note: RMSEA = Root-Mean-Square Error of Approximation; CFI = Comparative Fit Index; TLI = Tucker-Lewis Index. Model fit indices were evaluated such that acceptable fit was determined when CFI > 0.90, the TLI > 0.90 and RMSEA < 0.08 (Hu & Bentler, 1999).
