# Supplementary material for: Triangulating evidence for genetic and environmental components of associations between parental behaviours and aggressive behaviour in children
Source: Nat Commun. 2026 Jul 6;17:8340. doi: 10.1038/s41467-026-75095-5 (PMC13473577; doi:10.1038/s41467-026-75095-5)
Supplement: Supplementary file 2 [file 41467_2026_75095_MOESM2_ESM.pdf]

nature portfolio

Corresponding author(s): Joanna Bright

Last updated by author(s): Apr 28, 2026

Reporting Summary

Nature Portfolio wishes to improve the reproducibility of the work that we publish. This form provides structure for consistency and transparency in reporting. For further information on Nature Portfolio policies, see our Editorial Policies and the Editorial Policy Checklist.

Statistics

For all statistical analyses, confirm that the following items are present in the figure legend, table legend, main text, or Methods section.

n/a	Confirmed	
☐	☒	The exact sample size (n) for each experimental group/condition, given as a discrete number and unit of measurement
☒	☐	A statement on whether measurements were taken from distinct samples or whether the same sample was measured repeatedly
☐	☒	The statistical test(s) used AND whether they are one- or two-sided<br>*Only common tests should be described solely by name; describe more complex techniques in the Methods section.*
☐	☒	A description of all covariates tested
☐	☒	A description of any assumptions or corrections, such as tests of normality and adjustment for multiple comparisons
☐	☒	A full description of the statistical parameters including central tendency (e.g. means) or other basic estimates (e.g. regression coefficient) AND variation (e.g. standard deviation) or associated estimates of uncertainty (e.g. confidence intervals)
☐	☒	For null hypothesis testing, the test statistic (e.g. F, t, r) with confidence intervals, effect sizes, degrees of freedom and P value noted<br>*Give P values as exact values whenever suitable.*
☒	☐	For Bayesian analysis, information on the choice of priors and Markov chain Monte Carlo settings
☐	☒	For hierarchical and complex designs, identification of the appropriate level for tests and full reporting of outcomes
☐	☒	Estimates of effect sizes (e.g. Cohen's d, Pearson's r), indicating how they were calculated

Our web collection on statistics for biologists contains articles on many of the points above.

Software and code

Policy information about availability of computer code

Data collection	EGDS data collection detailed here: https://pmc.ncbi.nlm.nih.gov/articles/PMC3572752/<br>MoBa data collection detailed here: https://academic.oup.com/ije/article/45/2/382/2572643
Data analysis	R Studio, OpenMX, lavaan. Code used in the analysis of data is available at https://osf.io/dvxnc/files/osfstorage

For manuscripts utilizing custom algorithms or software that are central to the research but not yet described in published literature, software must be made available to editors and reviewers. We strongly encourage code deposition in a community repository (e.g. GitHub). See the Nature Portfolio guidelines for submitting code & software for further information.

Data

Policy information about availability of data

All manuscripts must include a data availability statement. This statement should provide the following information, where applicable:

- Accession codes, unique identifiers, or web links for publicly available datasets
- A description of any restrictions on data availability
- For clinical datasets or third party data, please ensure that the statement adheres to our policy

MoBa data is not publicly available because the consent given by the participants does not allow for data storage on an individual level in repositories or journals.

MoBa data can be accessed by application to the Regional Committee for Medical and Health Research Ethics in Norway and MoBa (https://www.fhi.no/en/ch/

studies/moba/for-forskere-artikler/research-and-data-access/).

Fully deidentified EGDS data has been uploaded to OSF and is available at https://doi.org/10.17605/OSF.IO/DVXNC.

Research involving human participants, their data, or biological material

Policy information about studies with human participants or human data. See also policy information about sex, gender (identity/presentation), and sexual orientation and race, ethnicity and racism.

Reporting on sex and gender	Main analyses focus on maternal reports as this was consistently available across both samples. Paternal reports were used in sensitivity anayses for the EGDS samples. Differences related to child sex assigned at birth was tested upon request from reviewers, and in main analyses regressed out of variables as a covariate.
Reporting on race, ethnicity, or other socially relevant groupings	N/A
Population characteristics	See below
Recruitment	EGDS recruitment process detailed here: https://pmc.ncbi.nlm.nih.gov/articles/PMC3572752/<br>MoBa recruitment process detailed here: https://academic.oup.com/ije/article/45/2/382/2572643
Ethics oversight	The establishment of MoBa and initial data collection was based on a license from the Norwegian Data Protection Agency and approval from The Regional Committees for Medical and Health Research Ethics. The MoBa cohort is currently regulated by the Norwegian Health Registry Act.<br><br>Ethical approval for EGDS data collection was obtained from the three institutional review boards for the universities involved in data collection, Penn State University, Oregon Social Learning Center, and George Washington University.<br><br>Data analysis was conducted on de-identified data at King's College London (KCL). At KCL, analysis of de-identified secondary data is exempt from the need for further ethical approval by the KCL Research Ethics Committee.

Note that full information on the approval of the study protocol must also be provided in the manuscript.

Field-specific reporting

Please select the one below that is the best fit for your research. If you are not sure, read the appropriate sections before making your selection.

☐ Life sciences ☒ Behavioural & social sciences ☐ Ecological, evolutionary & environmental sciences

For a reference copy of the document with all sections, see nature.com/documents/nr-reporting-summary-flat.pdf

Behavioural & social sciences study design

All studies must disclose on these points even when the disclosure is negative.

Study description	Quantitative, cross sectional family design in MoBa and longitudinal adoption design in EGDS
Research sample	This study uses samples from two existing datasets.<br><br>The Norwegian Mother, Father and Child Cohort Study (MoBa) is a population-based pregnancy cohort study conducted by the Norwegian Institute of Public Health. Participants were recruited from all over Norway between 1999-2008, with a recruitment rate of 41% of all pregnant women contacted during this time. Participants gave informed consent for themselves and their children, with questionnaires administered regularly over the following 20 years. The cohort includes approximately 114,500 children, 95,200 mothers and 75,200 fathers, with a mean maternal age at birth of 27.6 (SD=4.6)52. Mothers had on average 15.23 years of education (SD=2.27). The mean annual income after tax for this sample at first contact was 744,551 NOK (SD=45,396 NOK) which is significantly higher than the Norwegian population average, and converts to ~$74,00053. https://academic.oup.com/ije/article/45/2/382/2572643<br><br>The Early Growth and Development Study (EGDS) is a US prospective study following adopted children, and their adoptive and biological parents (N = 561 triads) recruited in two cohorts between 2000-2009, with regular assessments over the following 21 years. After receiving a complete description of their participation, all parents provided written informed consent for themselves, and adoptive parents consented for their child. Chi ldren were adopted within 3 months of birth, into non-biologically related families. Adopted children were 57% male. Adoptive mothers were an average of 37.8 years old (standard deviation = 5.50) at the time of the adopted child's birth, with median educational attainment at the college degree level. Adoptive families had a median annual income of $100,000-$125,000. To be consistent with the MoBa component of this study, we used mother reports of parenting and child outcomes. In sensitivity analyses, we reran analyses using paternal reports. https://pmc.ncbi.nlm.nih.gov/articles/PMC3572752/
Sampling strategy	MoBa: The sampling was opportunistic, due to limited initial funding. The recruitment commenced in a large hospital in the city of Bergen in the summer of 1999 and gradually expanded to include 50 of Norway's 52 hospitals with maternity units.<br><br>EGDS recruitment occurred between March 2003–January 2010 with the recruitment of adoption agencies into the study (N = 45

	agencies in 15 states). EGDS employs separate birth parent and adoptive family recruiters to ensure that project staff do not transfer information between members of the adoption triad. From Leve et al (2010) "Power analyses suggested that a sample of 331 triads would provide power well above 0.90 for detecting genetic and environmental main effects, power of 0.50–0.98 for detecting G×E interaction effects, and power of 0.78–0.93 for model testing in SEM, suggesting that the EGDS is sufficiently powered."
Data collection	EGDS data collection process detailed here: https://pmc.ncbi.nlm.nih.gov/articles/PMC3572752/. Assessments are ongoing, in 9-month intervals until the child reaches 3 years of age and in one-year intervals thereafter through age 9.<br>EGDS assessments include questionnaires, in-person interviews, and standardized testing for birth parents, adoptive parents, and children; diagnostic interviews with adoptive parents (about themselves and about the adopted child) and birth parents; observational interactions (mother-father, mother-child, father-child, and mother-father-child) for adoptive families; food and activity diaries for adoptive families; medical records for birth parents and the adopted child; DNA collection via buccal cells for adopted children, adoptive parents, and birth parents; diurnal cortisol measures for adopted children and birth parents; teacher questionnaires for children; and official arrest records for birth parents. The interviews include interviewer-administered questions, which create a context whereby the interviewer can establish rapport with the participant, and computer-assisted personal interviews (CAPI) that are completed privately by participants to facilitate confidentiality and honest responses. In-person assessments (adoptive family: age 9 months, 18 months, 27 months, and age 4.5, 6, 7, and 8 years; birth parents: 4- and 18-months postpartum and 4.5 years postpartum) last approximately 3–4 hrs each and are conducted in a location convenient for the participant, most often at home.<br><br>Brief telephone interviews (15 minutes) are conducted for birth and adoptive parents in between the primary in-person assessments and last approximately 15 minutes. These also serve as a means of maintaining contact and rapport with participants. Overall, 149 different assessment measures have been administered focusing on the primary theoretical model and aims. A full listing of study measures can be found within the study website: https://www.egdstudy.org/measures. THie present study used questionnaire data only.<br><br>MoBa collection process detailed here: https://academic.oup.com/ije/article/45/2/382/2572643. Data was collected via questionnaires, neuropsychological tests, samples, and clinical examinations. This study used questionnaire data only. Further detail on the measures used in each questionnaire can be found here: https://www.fhi.no/en/ch/studies/moba/for-forskere-artikler/viktige-dokumenter-for-moba-forskere/
Timing	MoBa: Participants were recruited from all over Norway from 1999-2008, with questionnaires administered regularly over the following 20 years.<br>EGDS: recruited participation in two cohorts between 2000-2009, with regular assessments over the following 21 years. Assessments are ongoing, in 9-month intervals until the child reaches 3 years of age and in one-year intervals thereafter through age 9.
Data exclusions	There are no exclusion criteria for participants in MoBa.<br><br>Five inclusion criteria were used in the EGDS: completion of a domestic adoption placement, placement of the baby within 3 months postpartum, placement within a nonrelative adoptive family, no known major medical conditions (e.g., extreme prematurity or extensive medical surgeries), and birth and adoptive parents were able to read or understand English at the eighth-grade level. All birth and adoptive families who met these inclusion criteria during the recruitment period were considered for enrollment.
Non-participation	We used existing samples in this study, therefore drop out/declining participation is not relevant to our study. MoBa participation rates are detailed here: https://academic.oup.com/ije/article/45/2/382/2572643 and EGDS here https://pmc.ncbi.nlm.nih.gov/articles/PMC3572752/
Randomization	This was not an experimental design and so randomization is not relevant. Covariates of interest were included in the models.

Reporting for specific materials, systems and methods

We require information from authors about some types of materials, experimental systems and methods used in many studies. Here, indicate whether each material, system or method listed is relevant to your study. If you are not sure if a list item applies to your research, read the appropriate section before selecting a response.

Materials & experimental systems

n/a	Involved in the study	
☒	☐	Antibodies
☒	☐	Eukaryotic cell lines
☒	☐	Palaeontology and archaeology
☒	☐	Animals and other organisms
☒	☐	Clinical data
☒	☐	Dual use research of concern
☒	☐	Plants

Methods

n/a	Involved in the study	
☒	☐	ChIP-seq
☒	☐	Flow cytometry
☒	☐	MRI-based neuroimaging

Plants

Seed stocks

Report on the source of all seed stocks or other plant material used. If applicable, state the seed stock centre and catalogue number. If plant specimens were collected from the field, describe the collection location, date and sampling procedures.

Novel plant genotypes

Describe the methods by which all novel plant genotypes were produced. This includes those generated by transgenic approaches, gene editing, chemical/radiation-based mutagenesis and hybridization. For transgenic lines, describe the transformation method, the number of independent lines analyzed and the generation upon which experiments were performed. For gene-edited lines, describe the editor used, the endogenous sequence targeted for editing, the targeting guide RNA sequence (if applicable) and how the editor was applied.

Authentication

Describe any authentication procedures for each seed stock used or novel genotype generated. Describe any experiments used to assess the effect of a mutation and, where applicable, how potential secondary effects (e.g. second site T-DNA insertions, mosiacism, off-target gene editing) were examined.
