# Supplementary material for: Triangulating evidence for genetic and environmental components of associations between parental behaviours and aggressive behaviour in children
Source: Nat Commun. 2026 Jul 6;17:8340. doi: 10.1038/s41467-026-75095-5 (PMC13473577; doi:10.1038/s41467-026-75095-5)
Supplement: Supplementary file 3 [file 41467_2026_75095_MOESM3_ESM.pdf]

nature portfolio

Peer Review File

Triangulating evidence for genetic and environmental components of associations between parental behaviours and aggressive behaviour in children

Corresponding Author: Ms Joanna Bright

This file contains all reviewer reports in order by version, followed by all author rebuttals in order by version.

Version 0:

Reviewer comments:

Reviewer #1

(Remarks to the Author)
Bright and colleagues examine bidirectional relationships between parenting and childhood aggression using two complementary family-based designs: a multiple children-of-twins/siblings approach in the Norwegian Mother, Father and Child Cohort Study (MoBa), and a longitudinal adoption design in the Early Growth and Development Study (EGDS). The study addresses a long-standing and important challenge in disentangling the direction of effects in parenting–child aggression behaviour associations while accounting for genetic confounding. The manuscript is well-written, and the presented analyses are rigorously conducted. The conclusions are largely well-justified, and the Discussion section is detailed and nuanced. Below, I list several specific suggestions that may further improve this work.

1. A key concern across both samples is the potential for shared method variance, which can inflate intergenerational associations when the same parent provides data on both parenting and child outcomes. In such cases, parents might unintentionally underreport negative parenting while overreporting child aggression to justify their behaviour. This issue is particularly relevant given the study's aim to distinguish the direction of causal influences between parenting and child aggression. The authors appropriately acknowledge this limitation, particularly in MoBa where only maternal reports are available. However, in EGDS, both maternal and paternal reports are available, offering an opportunity to mitigate this concern more directly. I recommend that the authors make fuller use of the paternal data in EGDS, by conducting sensitivity analyses in which i) child aggression is reported by fathers in mother-child dyads, ii) child aggression is reported by mothers in father-child dyads. These analyses would help assess the robustness of the findings to potential reporter bias.

2. One important limitation that is not currently addressed in the manuscript is the potential role of assortative mating. If mothers and fathers tend to mate based on similar genetically influenced traits, such as aggression or parenting behaviors, this can increase genetic similarity between parent and child beyond what is expected under random mating. As such, the decomposition of parent-child covariance into genetic and environmental components may overestimate the genetic pathway. Without accounting for assortative mating, estimates of genetic transmission may be inflated in the MCoTS analyses in MoBa, potentially overstating the role of shared genetics in parent–child associations. I would suggest that the authors acknowledge this limitation and briefly discuss how unmodeled assortative mating may have influenced their findings.

3. I understand that in the MCoTS analyses, adjustment for SES or parental psychopathology (e.g., depression) is not typically necessary, since shared family-level factors are captured within the shared environmental component. However, in EGDS, these variables are not automatically controlled for by the design and should be more carefully considered, given both are known to be associated with parenting behaviours and child aggression and may confound observed associations if not accounted for. I recommend that the authors either include SES and parental psychopathology as covariates in EGDS models or provide a clear rationale for their exclusion.

4. The parenting–child aggression association (in both parent-to-child and child-to-parent directions) may differ by child sex, as previous research has identified sex-specific patterns in both aggression and parenting responses. It would strengthen the study to test for moderation by child sex, or, where possible, conduct sex-stratified analyses. If these analyses are not feasible due to power constraints, a brief discussion of this point would be useful.

Some minor comments
- The Alabama Parenting Questionnaire (APQ) includes five domains of parenting behaviours: parental involvement, positive parenting, poor monitoring/supervision, inconsistent discipline, and corporal punishment. However, only positive parenting and inconsistent discipline were included in the current analyses. Please justify the decision to include only these two domains and explain why the others were excluded.

- Please clarify the type of reliability coefficient reported for parenting and aggression measures (Cronbach's alpha?).

- The authors report residualizing covariates (e.g., maternal age, child year of birth, parity, child sex) from both exposure and outcome variables prior to analysis. This approach can introduce bias vs. including covariates properly within the model (or by regressing the covariates out of both the exposure and the outcome) Please clarify the rationale for this approach.

- I was not able to find key sample characteristics (e.g., sample size, maternal age, SES/education, child age and sex distribution) or a descriptive statistics table for the main variables in either MoBa or EGDS. For clarity and transparency, these details should be clearly reported across both cohorts, or per analytic model.

- The sentence "the genes involved in the inconsistent discipline/less positive parenting in parents are expressed as aggressive behaviours in children" sounds deterministic. Additionally, the phrase "genes ... are expressed" has a specific meaning in molecular biology (gene expression). I suggest rephrasing it.

(Remarks on code availability)

Reviewer #2

(Remarks to the Author)
This is a well-written paper examining associations between parenting behaviors and child aggression in two samples of parents and children. Although the manuscript has strengths, including the use of multiple samples to test hypotheses, I offer the following points for consideration:

1. Both samples included in this study are quite old (i.e., data collected between 1998-2008 and 2000-2009). Given societal/historical changes that may have occurred in the last 15-20 years, it may be worth at least acknowledging generalizability of these findings to more recent times as a limitation in the discussion section.

2. It would be helpful if more demographic information about the samples was presented in the methods section to help with interpretation and understanding the potential generalizability of findings (e.g., sex of parents and children in the studies, ages, etc.).

3. Although the authors acknowledge in the limitations that all measures were parent-report, it seems also important to acknowledge that all the measures in this study were very short questionnaires. Given that there are many studies using more mixed-methods and rigorous assessments of parenting and/or child behavior, this seems like a significant limitation of this methodology.

4. Very limited information on the study methodology is presented in the methods section. It would be helpful to provide additional information, such as specifics on which reporter (e.g., mother, father, other) completed each questionnaire and to provide sample items for each tool.

5. Although the authors controlled for some demographic information, it seems that it would be important to also account for some indices of family socioeconomic status as well.

6. In its current form, the results section is a little hard to follow. It may be helpful to lay them out so they clearly describe what the authors found in alignment with the research questions and hypotheses.

(Remarks on code availability)

Reviewer #3

(Remarks to the Author)
This study by Bright et al. used two family-based samples to examine the direction of and causality underlying the association between parenting behaviors and child aggression. The MoBa and EGDS samples had comparable measures of parenting and aggression but different designs to allow for triangulation. The association between positive parenting and lower child aggression was mainly driven by shared genetics. Interestingly, while in both samples there was some degree of shared genetics between inconsistent parenting and child aggression, there was converging evidence that child aggression resulted in more inconsistent parenting behavior. The study is well written, methodology and analysis are sound, and the study is interesting for the broader literature.

- In the abstract, it would be good to better explain which family-based designs were used and how they complement each

other. Now this is unclear and difficult for the reader to find out how they even take genetics into account and what this means for triangulation.
- In the abstract and discussion, it would be interesting to read more about how these findings fit in the current body of literature. Especially with regards to other studies that take genetics into account, such as the aggression/antisocial GWASes, PGS studies, parent-offspring trio designsn, or perhaps even MR studies. This would support the authors' aim of triangulation.
- In terms of clinical implications, what would the authors advise for interventions? If replicated and true, this could mean that professionals need to target children and not necessarily the parents in order to improve parent and/or reduce child aggression. This would be new as most treatment guidelines in child & adolescent psychiatry/psychology (the kids of these samples were aged between 5-8 years olds) advise to start with parents.
- I think that the short measures of aggression (5 items only in MoBa!) and parenting (6 items only) is quite a big limitation. Especially considering the strong conclusions inferred from the results. I realize this is addressed in the Discussion, but the fact that in both MoBa and EGDS results were in the same direction does not mean this will hold in other samples (with better measures). This does have important implications for your findings, and how will this be translated into practice? I'm sure child (mental) health) professionals need a bit more than 5+6 items of child/parent behaviors to help their patients, so I'd advise to put this more nuanced in your conclusions and implications for (clinical) practice.
- Following from this, I think that the final bit of the Abstract could also be revised.
- Some descriptive characteristics and N's of the MCoTS model sample would be appreciated (for EGDS these are presented in supp tables 4 and 5).

I hope the above points help to improve the manuscript.

(Remarks on code availability)

Reviewer #4

(Remarks to the Author)
Note: This review was requested to specifically focus on the SEM / twin study methodology, so this review will focus on those aspects.

This study presents a twin study and structural equation modeling (SEM) analysis of parenting behaviors and child aggression. This approach provides a unique opportunity to estimate the impact of genetics vs shared environment on psychological traits. The modeling was generally very thorough, and a lot of detail was provided in terms of modeling strategy and overall interpretation of best fitting models. An additional strength was the use of validated measures. Yet another strength was the inclusion of an adoption cohort with similar quantitative measures to get a better sense for the impact of shared environment. However, for a high impact generalist journal like this one, there are a few areas that need to be better addressed, as noted below, in no particular order.

The conclusions and impact of this work need to be more clearly and strongly articulated. The abstract cites an estimate (47%) of the proportion of effect due to shared genetics. It would be good if the Discussion also led off with such quantitative conclusions. A summary table of the major findings would be extremely helpful to the reader. While the SEM figures are important, the readers of this journal and in the field will likely want to see overall quantitative conclusions in a more palatable format. This includes the proportion of variation due to shared environment, directionality of that effect, across all of the various models and traits assessed, etc. The results section talks a lot about which paths were retained, but does not quantify the strength of those effects – that gets at importance and impact. It is worth considering minimizing the number of path diagrams in the main text and putting emphasis on the most important ones, possibly the final models, and add tables that summarize the quantitative results more clearly.

For the twin study data, it is not stated whether it was validated whether the children were monozygotic vs dizygotic twins – this is important in disentangling genetic effects (additive vs dominance variance).

A Table 1 of the subjects included in the analyses must be included. This should include demographics, number of families, number of MZ vs DZ twins, siblings, distributions of variables, etc.

It says that confirmatory factor analysis was used to construct the factors – from the figures, it isn’t clear how these were parametrized / how well the factors were identified. Was a variance set to 1, or what else was done to identify the model?

One strength of this study was the inclusion of an adoption cohort to better understand the impact of shared environment. However, there are a couple of limitations here that need to be addressed. First, this cohort comes from a different country – what is the impact on different cultural norms and other societal factors on shared environment? This is somewhat addressed in the limitations section but deserves more emphasis. Second, the summary table suggested above would be helpful here – while the Discussion says the conclusions are similar, it is really hard to see that without a quantitative head-to-head comparison.

Minor comments:
Line 102 – the word “avuncular” is used here. I don’t think this is the word you mean, since this refers to aunt-uncle / niece-nephew relationships.

Supplemental Table 1: Consider either providing supplemental methods and/or a detailed footnote to interpret these parameters. The reader unfamiliar with SEM will not know how CFI, TLI, and RMSEA are similar/different to the p-values and regression model fit statistics they are used to seeing.

Supplemental Table 3: please indicate in the footnote what is meant by the bold font.

(Remarks on code availability)
Code was not provided, nor do I think that it is essential for this.

Version 1:

Reviewer comments:

Reviewer #1

(Remarks to the Author)
I am pleased to review the revised manuscript. The authors have addressed all of my previous comments. The revisions have strengthened the manuscript, improving its clarity, methodological rigor, and overall contribution to the field. I have no further comments or suggestions.

Reviewer #2

(Remarks to the Author)
The authors' responses to reviewer comments seem thorough and sufficient. I have nothing further to add.

Reviewer #3

(Remarks to the Author)
Thank you for addressing my comments, I am satisfied with the response to my questions.

Reviewer #4

(Remarks to the Author)
Thank you for so thoroughly responding to the original critique. I have no additional comments.

Open Access This Peer Review File is licensed under a Creative Commons Attribution 4.0 International License, which permits use, sharing, adaptation, distribution and reproduction in any medium or format, as long as you give appropriate credit to the original author(s) and the source, provide a link to the Creative Commons license, and indicate if changes were made.
In cases where reviewers are anonymous, credit should be given to 'Anonymous Referee' and the source.
The images or other third party material in this Peer Review File are included in the article's Creative Commons license,

unless indicated otherwise in a credit line to the material. If material is not included in the article's Creative Commons license and your intended use is not permitted by statutory regulation or exceeds the permitted use, you will need to obtain permission directly from the copyright holder.
To view a copy of this license, visit https://creativecommons.org/licenses/by/4.0/

Key:
Reviewer comments = red
Author responses = black
Revisions to the manuscript text = blue

Reviewer #1 (Remarks to the Author):

Bright and colleagues examine bidirectional relationships between parenting and childhood aggression using two complementary family-based designs: a multiple children-of-twins/siblings approach in the Norwegian Mother, Father and Child Cohort Study (MoBa), and a longitudinal adoption design in the Early Growth and Development Study (EGDS). The study addresses a long-standing and important challenge in disentangling the direction of effects in parenting–child aggression behaviour associations while accounting for genetic confounding. The manuscript is well-written, and the presented analyses are rigorously conducted. The conclusions are largely well-justified, and the Discussion section is detailed and nuanced. Below, I list several specific suggestions that may further improve this work.

1. A key concern across both samples is the potential for shared method variance, which can inflate intergenerational associations when the same parent provides data on both parenting and child outcomes. In such cases, parents might unintentionally underreport negative parenting while overreporting child aggression to justify their behaviour. This issue is particularly relevant given the study's aim to distinguish the direction of causal influences between parenting and child aggression. The authors appropriately acknowledge this limitation, particularly in MoBa where only maternal reports are available. However, in EGDS, both maternal and paternal reports are available, offering an opportunity to mitigate this concern more directly. I recommend that the authors make fuller use of the paternal data in EGDS, by conducting sensitivity analyses in which i) child aggression is reported by fathers in mother-child dyads, ii) child aggression is reported by mothers in father-child dyads. These analyses would help assess the robustness of the findings to potential reporter bias.

We thank the reviewer for their positive comments and for this suggestion. We ran the suggested analyses and adjusted the manuscript as follows.

Methods: *Line 372-378*
In EGDS we had access to paternal reports of parenting and child outcomes. We made use of the second reporter to conduct sensitivity analyses using i) paternal reports of parenting behaviours and child aggression; ii) paternal reports of child aggression in mother-child dyads, iii) maternal reports of child aggression father-child dyads. Analyses using paternal reports for both parent and child variables were preregistered. Analyses ii) and iii) were run following the suggestion of an anonymous reviewer, and so were not preregistered.

Results: *Line 531-549*
Sensitivity analyses: Making use of paternal reports
Whilst only maternal reports were used in the main analyses to allow for comparison across samples, we carried out sensitivity analyses in EGDS to see whether there was a similar pattern of results when using paternal reports (available for an average of 87% of children at each wave; see Supplementary Table 12 for fit statistics). We ran sensitivity analyses using i) paternal reports of parenting behaviours and child aggression; ii) paternal reports of child aggression in mother-child dyads, iii) maternal reports of child aggression in father-child dyads.

For analyses using paternal reports for both parenting and child aggression (see Supplementary Table 11 for main results), we found significant within-timepoint paths from parent-to-child (estimated at 0.35) and child-to-parent (estimated at 1.70) for the relationship between inconsistent discipline and child aggressive behaviour. However, there were no significant cross lags. For analyses using positive parenting, there were no significant associations between paternal reports of positive parenting and child aggression either within or across time. There were no significant paths when using father reported child aggression in mother-child dyads or mother reported child aggression in father-child dyads.

Discussion: *Line 691-711*
Research has shown differential parent-child associations dependent on the gender of the parent[7,52], which indeed was what we found in EGDS where there were significant parent-to-child within time paths for inconsistent discipline. In EGDS sensitivity analyses, we compared paternal reports of child aggression in mother-child dyads and vice versa. In these analyses we found no evidence of significant parent-to-child or child-to-parent paths, which could suggest that using the same reporter for both parent and child behaviours led to inflated associations. It is known that using the same reporters for both parent and child reports may bias responses[53], and so the inclusion of multiple reporters for each observation of behaviour would allow researchers to control for some of these biases. Alternatively, mothers and fathers may pick up different aspects of parenting and child aggression, in ways that are unrelated. For example, fathers may rate children as less aggressive than mothers, or may report their parenting as less consistent, which may reduce associations with mother-reported variables. We note that previous work in our group[54] has shown that a polygenic score for externalising is as predictive of parent-reported child externalising behaviour as it is of child-reported and teacher-reported externalising, independently of parental indirect genetic effects, even though parent, teacher and child-report externalising are only moderately correlated. This demonstrates that regardless of the reporter, measures of externalising do index a child's genetically influenced behaviour; thus, we can assume that parent-report is not all bias and is truly reflective of a child's behaviour.

2. One important limitation that is not currently addressed in the manuscript is the potential role of assortative mating. If mothers and fathers tend to mate based on similar genetically influenced traits, such as aggression or parenting behaviors, this can increase genetic similarity between parent and child beyond what is expected under random mating. As such, the decomposition of parent-child covariance into genetic and environmental components may overestimate the genetic pathway. Without accounting for assortative mating, estimates of genetic transmission may be inflated in the MCoTS analyses in MoBa, potentially overstating the role of shared genetics in parent–child associations. I would suggest that the authors acknowledge this limitation and briefly discuss how unmodeled assortative mating may have influenced their findings.

We agree that this limitation is important to highlight. We have added the following text to the discussion to recognise the potential influence of assortative mating in our MCoTS models.

Discussion: *Line 713-728*
Further, by including only one parent in our MCoTS analyses, we could not control for the possible effects of assortative mating, the phenomenon where people choose partners who are like them for specific behaviors, i.e. are "phenotypically similar". The implications of assortative mating for decomposing intergenerational relationships are complex, with alternative explanations for coparent similarities such as partners being alike on social or cultural factors, or become more phenotypically

similar as they spend more time together[27]. There is evidence for assortative mating on aggressive or violent behaviours[58], thus future research could incorporate coparent data and/or observational measures of parent-child interaction into MCoTS models to elucidate potential biases from assortative mating on the genetic and environmental components of the relationship between parenting behaviours and child aggression. That being said, assortative mating does not similarly impact the adoption design: in adoption studies, associations between adoptive parent and child remain attributable to non-genetic causes regardless of whether birth or adoptive parents mate selectively. That our MCoTS and EGDS analyses arrive at the same conclusions would suggest that assortative mating is not biasing our findings.

3. I understand that in the MCoTS analyses, adjustment for SES or parental psychopathology (e.g., depression) is not typically necessary, since shared family-level factors are captured within the shared environmental component. However, in EGDS, these variables are not automatically controlled for by the design and should be more carefully considered, given both are known to be associated with parenting behaviours and child aggression and may confound observed associations if not accounted for. I recommend that the authors either include SES and parental psychopathology as covariates in EGDS models or provide a clear rationale for their exclusion.

Thank you for this suggestion. We agree with the inclusion of SES and so in the EGDS analyses we have included SES at age 7 (when we were able to compute a full index including income and education) as a covariate and highlighted this change in the methods (line 258). However, we chose not to include a measure of maternal psychopathology, as it could be a mediator. There is reasonable doubt that parental mental health is only a confounder. For example, a study by Bjørndal et al. (2024) found child indirect genetic effects on maternal mental health with no reverse causality https://www.nature.com/articles/s44220-024-00207-3. Hence, we believe that including maternal psychopathology into the models changes the form of the research question beyond the scope of our original plans.

4. The parenting–child aggression association (in both parent-to-child and child-to-parent directions) may differ by child sex, as previous research has identified sex-specific patterns in both aggression and parenting responses. It would strengthen the study to test for moderation by child sex, or, where possible, conduct sex-stratified analyses. If these analyses are not feasible due to power constraints, a brief discussion of this point would be useful.

We appreciate this suggestion to strengthen the analysis. We tested for sex effects in both samples. In the MCoTS analyses, we ran sex-stratified sensitivity analyses, by building family structures around only boys or girls in the child generation. and running the MCoTS models for mothers reporting on parenting their male children only, and then separately for mothers reporting on parenting their female children. As these were not nested models, we could not compare model fit, however we tested sex differences by comparing confidence intervals for each variance component across the two sexes. Male and female variance components extracted for the models had overlapping confidence intervals, and so we concluded that there were no significant sex differences. The wide confidence intervals and non-significant A estimates also suggest that these models were underpowered due to splitting the sample in half.

For EGDS, we tested sex differences by incorporating child sex as a categorical grouping variable in lavaan. Multi-group models were then estimated by specifying the sex variable in the group argument. We then ran one model with parameters allowed to be freely estimated across the two sexes, and then a second where the paths of interest (parent-to-child and child-to-parent) were

constrained to be equal across sexes. As the models were nested, we could compare model fit. We found non-significant difference in fit between the unconstrained and constrained models, which suggests no sex differences in the paths of interest for either parenting dimension.

These analyses, as detailed above, have been added to the methods section (lines 307-314 for MoBa analyses, lines 379-387 for EGDS analyses). Results are discussed in lines 515-528 and in Supplementary Tables 7, 8, 11 & 13.

Some minor comments

- The Alabama Parenting Questionnaire (APQ) includes five domains of parenting behaviours: parental involvement, positive parenting, poor monitoring/supervision, inconsistent discipline, and corporal punishment. However, only positive parenting and inconsistent discipline were included in the current analyses. Please justify the decision to include only these two domains and explain why the others were excluded.

We appreciate the opportunity to clarify this decision. Only these domains were available in both samples, and as a main aim of this paper was to attempt triangulation, we kept the paper focused on the overlapping dimensions. We have added text to explain this in more detail on lines 207-212.

Please clarify the type of reliability coefficient reported for parenting and aggression measures (Cronbach's alpha?).

We have added clarification that the reliability coefficient was Cronbach's α on page 10.

- The authors report residualizing covariates (e.g., maternal age, child year of birth, parity, child sex) from both exposure and outcome variables prior to analysis. This approach can introduce bias vs. including covariates properly within the model (or by regressing the covariates out of both the exposure and the outcome). Please clarify the rationale for this approach.

Thank you for flagging this potential source of bias. We have considered the alternative approach to controlling for covariates in our models. However, given the complexity of these models, particularly the MCoTS model, regressing out the covariates from the exposure and outcome variables prior to analysis allowed us to simplify the final models as much as possible and to set up our models using the two different datasets in as comparable a way as possible. In MoBa, parenting and child outcome variables were regressed on maternal age, child year of birth, parity, and child sex-assigned-at-birth. In EGDS, all parent and child variables were regressed on an index of socioeconomic status (SES; created from adoptive parents' education level and income when the child was 7), child sex-assigned-at-birth, adoption openness (a measure of contact between birth and adoptive families) and maternal age.

- I was not able to find key sample characteristics (e.g., sample size, maternal age, SES/education, child age and sex distribution) or a descriptive statistics table for the main variables in either MoBa or EGDS. For clarity and transparency, these details should be clearly reported across both cohorts, or per analytic model.

We thank the reviewers for highlighting this omission. We have now included key sample characteristics in the text as shown on lines 165-203. In the supplementary material, we have also included a table of *N*s in parent and child generation for the MoBa sample (Supplementary Table 1), and Supplementary

Table 2 with descriptive statistics for the variables of interest in MoBa, to compliment the descriptive statistics for EGDS in Supplementary Table 9.

The sentence "the genes involved in the inconsistent discipline/less positive parenting in parents are expressed as aggressive behaviours in children" sounds deterministic. Additionally, the phrase "genes ... are expressed" has a specific meaning in molecular biology (gene expression). I suggest rephrasing it.

Thank you to the reviewer for catching this awkward wording. We agree and have edited the text accordingly.

Discussion: *Line 562-565*
Together, these findings suggest that the genetic factors associated with higher levels of inconsistent discipline/lower levels of positive parenting in parents are also associated with higher levels aggressive behaviours in children.

Reviewer #2 (Remarks to the Author):
This is a well-written paper examining associations between parenting behaviors and child aggression in two samples of parents and children. Although the manuscript has strengths, including the use of multiple samples to test hypotheses, I offer the following points for consideration:

1. Both samples included in this study are quite old (i.e., data collected between 1998-2008 and 2000-2009). Given societal/historical changes that may have occurred in the last 15-20 years, it may be worth at least acknowledging generalizability of these findings to more recent times as a limitation in the discussion section.

Thank you for your support of our manuscript and for raising this point around generalisability. We have clarified in the text that the dates 1998-2008/2000-2009 were timeframes for recruitment, and that data collection happened over the following 20 years (Line 166-167 for MoBa and Line 188 for EGDS). We also added a point to the discussion to highlight the limitation around generational differences:

Discussion: *Line 747-754*
Both studies began recruitment in the early 2000's, thus data used in this study was collected between 10-23 years ago. This means that children included in these studies are almost a generation older than children being born today. Socially "normative" parenting practices are thought to change across generations[65,66], as does understanding of "typical" child behaviours[67]. It is possible that associations between parents and children born in the early 2000s may look different to those born today. If true, this could have implications for how our findings may translate to interventions or clinical practice in today's children and parents.

2. It would be helpful if more demographic information about the samples was presented in the methods section to help with interpretation and understanding the potential generalizability of findings (e.g., sex of parents and children in the studies, ages, etc.).

Thank you for this suggestion to correct this omission on our part. We have included key sample characteristics in the text as shown on lines 165-203. In the supplementary material, we have also included a table of *N*s in parent and child generation for the MoBa sample (Supplementary Table 1),

and Supplementary Table 2 with descriptive statistics for the variables of interest in MoBa, to compliment the descriptive statistics for EGDS in Supplementary Table 9.

3. Although the authors acknowledge in the limitations that all measures were parent-report, it seems also important to acknowledge that all the measures in this study were very short questionnaires. Given that there are many studies using more mixed-methods and rigorous assessments of parenting and/or child behavior, this seems like a significant limitation of this methodology.

We agree that the length of the measures is a common limitation of population-level data collection such as in MoBa. We have edited the discussion to reflect this.

Discussion: *Line 676-685*
Whilst the measures used by both samples are rigorously validated, these subscales may only capture rather narrow dimensions of parenting and child behaviours. Therefore, used alone they may not fully translate to clinical practice or parent-child interventions. For example, the APQ is a short measure, with only 6 items, which may limit how well it captures complex parenting dynamics. It is important for future research to continue the triangulation approach presented here with methods that can incorporate more in-depth assessments of parenting and child behaviors (e.g., observations of parenting, teacher and youth reports of child behaviour), or use mixed methods approaches which are able to incorporate more nuanced aspects of parent-child relationships.

4. Very limited information on the study methodology is presented in the methods section. It would be helpful to provide additional information, such as specifics on which reporter (e.g., mother, father, other) completed each questionnaire and to provide sample items for each tool.

Thank you for flagging this gap in our description of the study methodology. We have updated the methods (Lines 215-229) to make it clear that all main analyses for both the MoBa and EGDS samples used maternal reports, with paternal reports used in the sensitivity analyses in EGDS. We also included sample items from the CBCL (lines 216-217).

5. Although the authors controlled for some demographic information, it seems that it would be important to also account for some indices of family socioeconomic status as well.

Thank you for this suggestion. We agree with the inclusion of SES, and so in the EGDS analyses, we have included SES at age 7 (when we were able to compute a full index including income and education) as a covariate and highlighted this change in the methods (line 258).

6. In its current form, the results section is a little hard to follow. It may be helpful to lay them out so they clearly describe what the authors found in alignment with the research questions and hypotheses.

We appreciate the feedback on a more readable layout for the results. We have restructured the results section to align with the research questions, first showing results for inconsistent parenting and child aggression across the two samples, and then displaying results for positive parenting and inconsistent discipline. We have also added a qualitative summary table (Table 1) which highlights the triangulation across samples and Tables 3 & 4 which highlight the main effects for the final models in each sample.

Reviewer #3 (Remarks to the Author):

This study by Bright et al. used two family-based samples to examine the direction of and causality underlying the association between parenting behaviors and child aggression. The MoBa and EGDS samples had comparable measures of parenting and aggression but different designs to allow for triangulation. The association between positive parenting and lower child aggression was mainly driven by shared genetics. Interestingly, while in both samples there was some degree of shared genetics between inconsistent parenting and child aggression, there was converging evidence that child aggression resulted in more inconsistent parenting behavior. The study is well written, methodology and analysis are sound, and the study is interesting for the broader literature.

- In the abstract, it would be good to better explain which family-based designs were used and how they complement each other. Now this is unclear and difficult for the reader to find out how they even take genetics into account and what this means for triangulation.

We thank the reviewer for their positive response to our manuscript and for this feedback which highlighted a lack of clarity in our abstract. We have updated the abstract to clarify these models, whilst observing the editorial guidelines' word count limit.

Abstract: *Line 35-48*

Parenting behaviours are often linked to childhood aggression, but many studies fail to account for shared genetics or test directionality. Using family-based designs, we controlled for genetic confounding and examined bidirectional effects across two samples. First, we applied a children-of-twins/siblings design in the Norwegian Mother Father and Child Cohort Study to separate genetic and environmental components of parent-child associations. Second, we used longitudinal models in the USA-based Early Growth and Development Study, an adoption sample enabling estimation of parent-child associations without genetic confounding. Analyses were pre-registered except where noted.

Across both studies, we found evidence for genetic and environmental transmission between parenting and child aggression, including significant child-to-parent effects on inconsistent discipline. These findings build on the growing body of literature showing genetic influences on both aggression and parenting behaviours, whilst underscoring the value of triangulation. Future research exploring interventions should consider child behaviour as a driver of parental discipline.

- In the abstract and discussion, it would be interesting to read more about how these findings fit in the current body of literature. Especially with regards to other studies that take genetics into account, such as the aggression/antisocial GWASes, PGS studies, parent-offspring trio designsn, or perhaps even MR studies. This would support the authors' aim of triangulation.

Thank you for these suggestions to incorporate more discussion of how our work fits within the current literature. We have added to our discussion to highlight where our findings fit with GWAS, polygenic score analysis and twin designs. We also edited the abstract (line 45) to briefly reflect these points.

Discussion: *Line 585-598*

Future research should investigate whether there is overlap between the genetic underpinnings of parenting dimensions and child outcomes, given that our findings suggest a shared genetic liability for child aggressive behaviours and inconsistent discipline/positive involvement from parents. There is a genetic component to an individual's risk for aggressive behaviour or conduct-related problems. A

genome-wide association study of general liability to externalising behaviours found that the resultant polygenic score predicted 2.0-2.3% of the variance in aggression, 1.2-3.1% of the variance in rule-breaking behaviour45 and up to 3.4% of the variance in child behaviour problems[46,47]. Intergenerational research has shown that associations between polygenic scores for parental externalising and risk-taking and child conduct problems were due to direct genetic transmission[48]. Twin designs have shown genetic influences on parenting behaviours, both at the parent level, and the level of the child who is receiving the parenting[49], suggestive evocative effects of the child genotype on the parenting they receive.

- In terms of clinical implications, what would the authors advise for interventions? If replicated and true, this could mean that professionals need to target children and not necessarily the parents in order to improve parent and/or reduce child aggression. This would be new as most treatment guidelines in child & adolescent psychiatry/psychology (the kids of these samples were aged between 5-8 years olds) advise to start with parents.

- I think that the short measures of aggression (5 items only in MoBa!) and parenting (6 items only) is quite a big limitation. Especially considering the strong conclusions inferred from the results. I realize this is addressed in the Discussion, but the fact that in both MoBa and EGDS results were in the same direction does not mean this will hold in other samples (with better measures). This does have important implications for your findings, and how will this be translated into practice? I'm sure child (mental) health) professionals need a bit more than 5+6 items of child/parent behaviors to help their patients, so I'd advise to put this more nuanced in your conclusions and implications for (clinical) practice.

- Following from this, I think that the final bit of the Abstract could also be revised.

We thank the reviewer for this insight into translatability for clinicians. We have responded to the three points together in the text, with edits made to line 46 in the abstract and a section on “Implications for parent-child interventions and clinical practice” added to the discussion (see below). We believe that whilst the findings suggest child-driven effects, this does not have to mean all interventions no longer start with children – instead interventions may support parents to understand their children’s behaviours more clearly.

<u>Discussion</u> *Line 643-654*
Our findings suggest that clinicians working with children should recognise that childhood aggression can drive inconsistent discipline. Thus, interventions for childhood aggression that are focused only on consistent discipline by parents may be insufficient. It may be beneficial to explore and additionally target other familial, environmental and individual factors associated with aggressive behaviours in the child. This research hints at potential causal links for parent-child behaviour patterns and evidence for the role of genetics in associations. However, measures of single domains of parent and child behaviours do not capture the complex nature of everyday parenting that interventions and clinical practice must consider. Hence, more research needs to be done to build a holistic picture of how day-to-day parenting interactions develop, and to disentangle the genetic and causal links between various domains of parenting and aggressive behaviour in children.

- Some descriptive characteristics and N's of the MCoTS model sample would be appreciated (for EGDS these are presented in supp tables 4 and 5). I hope the above points help to improve the manuscript.

Thank you for highlighting the omission of MCoTS descriptive characteristics. We have included key sample characteristics in the text as shown on lines 165-203. In the supplementary material, we have also included a table of *N*s in parent and child generation for the MoBa sample (Supplementary Table 1), and Supplementary Table 2 with descriptive statistics for the variables of interest in MoBa, to compliment the descriptive statistics for EGDS in Supplementary Table 9.

Reviewer #4 (Remarks to the Author):

Note: This review was requested to specifically focus on the SEM / twin study methodology, so this review will focus on those aspects.

This study presents a twin study and structural equation modeling (SEM) analysis of parenting behaviors and child aggression. This approach provides a unique opportunity to estimate the impact of genetics vs shared environment on psychological traits. The modeling was generally very thorough, and a lot of detail was provided in terms of modeling strategy and overall interpretation of best fitting models. An additional strength was the use of validated measures. Yet another strength was the inclusion of an adoption cohort with similar quantitative measures to get a better sense for the impact of shared environment. However, for a high impact generalist journal like this one, there are a few areas that need to be better addressed, as noted below, in no particular order.

The conclusions and impact of this work need to be more clearly and strongly articulated. The abstract cites an estimate (47%) of the proportion of effect due to shared genetics. It would be good if the Discussion also led off with such quantitative conclusions.

Thank you for your insightful comments regarding our SEM/twin methodology. We have edited the paragraph about genetic associations to include more quantitative conclusions.

Discussion: *Line 558-565*
Using the multiple-children-of-twins/siblings designs in the Norwegian MoBa study, we found that 47% of the association between inconsistent parenting and child aggression was explained by shared genetics. Further, the negative association between positive parenting and child aggression was completely (100%) explained by shared genetics. Together, these findings suggest that the genetic factors associated with higher levels of inconsistent discipline/lower levels of positive parenting in parents are also associated with higher levels aggressive behaviours in children.

A summary table of the major findings would be extremely helpful to the reader. While the SEM figures are important, the readers of this journal and in the field will likely want to see overall quantitative conclusions in a more palatable format. This includes the proportion of variation due to shared environment, directionality of that effect, across all of the various models and traits assessed, etc. The results section talks a lot about which paths were retained, but does not quantify the strength of those effects – that gets at importance and impact. It is worth considering minimizing the number of path diagrams in the main text and putting emphasis on the most important ones, possibly the final models, and add tables that summarize the quantitative results more clearly.

We thank the reviewer for these helpful suggestions for making the results more palatable. In the results section, we have added a qualitative summary table (Table 1) which highlights the triangulation across samples and summary tables (Tables 3 & 4) for each analysis design to lay out overall quantitative

results. We moved the path diagram for the unidirectional MCoTS model for inconsistent discipline & child aggression to the supplements (Supplementary Figure 3) so that we only report the direction-of-causation model in the main text. We moved the random intercept cross lagged panel model figures to the supplements (Supplementary Figure 5 & 6) to avoid too many figures in the main text, as they showed very similar patterns of results to the autoregressive bidirectional models.

For the twin study data, it is not stated whether it was validated whether the children were monozygotic vs dizygotic twins – this is important in disentangling genetic effects (additive vs dominance variance).

Thank you for highlighting this omission. We have added text to clarify how zygosity of twins in the child generation were validated (shown below). To be clear, the focal twin relationships are in the parent generation, although there were also a few twin pairs in the offspring generation (twins born to twins).

Methods: *Line 178-181*
We extracted extended families from MoBa, which comprised monozygotic (MZ) and dizygotic (DZ) twins, siblings, half-siblings and cousins in both the parent and child generation. Zygosity of twins was determined based on genotyping, questionnaires, and linkage to the Norwegian Twin Registry.

A Table 1 of the subjects included in the analyses must be included. This should include demographics, number of families, number of MZ vs DZ twins, siblings, distributions of variables, etc.

We appreciate the reviewer calling attention to the missing table of subject demographics. We have included Supplementary Table 1 as an overview of pedigree structures and sample sizes used in the MoBa study. This includes the number and type of extended family members. We have included key sample characteristics in the text as shown on lines 165-203. We have also included Supplementary Table 2 with descriptive statistics for the variables of interest in MoBa, to compliment the descriptive statistics for EGDS in Supplementary Table 9.

It says that confirmatory factor analysis was used to construct the factors – from the figures, it isn't clear how these were parametrized / how well the factors were identified. Was a variance set to 1, or what else was done to identify the model?

Thank you for highlighting this lack of clarity. We added Supplementary Table 4 to describe parameter estimates for each factor and model fit statistics in the Supplementary materials. We have added the following information to the methods to clarify how the factors were parametrized.

Methods: *Line 248-251*
We specified one factor per phenotype for inconsistent discipline (3 items loading onto the factor), positive parenting (3 items loading onto the factor) and child aggressive behaviours (5 items loading onto the factor). The factor loading of the first indicator for each phenotype was fixed to 1. The other factor loadings were freely estimated.

One strength of this study was the inclusion of an adoption cohort to better understand the impact of shared environment. However, there are a couple of limitations here that need to be addressed. First, this cohort comes from a different country – what is the impact on different cultural norms and other societal factors on shared environment? This is somewhat addressed in the limitations section but deserves more emphasis.

We thank the reviewer for highlighting this potential limitation. Whilst we agree that different social norms across countries can indeed influence how parents might respond to questions about parenting and child outcomes, we note that we found coherent patterns of results in these two samples for two distinct forms of parenting. Indeed, the fact that we triangulated using distinct methods in distinct samples in distinct countries is a strength of this analysis.

Discussion: *Line 739-747*
Whilst both samples are similarly skewed to higher socioeconomic advantage, they are also from different countries, with different cultural and societal factors. Norway and the US may have slightly different cultural norms when it comes to child aggression and parenting practices, and these norms could impact how parents responded to the measures included here. However, the fact that results and conclusions across these two samples were the same for two distinct forms of parenting strongly suggests that these differences in norms do not translate to differences in mechanisms underlying parent-child associations in North American and Norwegian contexts.

Second, the summary table suggested above would be helpful here – while the Discussion says the conclusions are similar, it is really hard to see that without a quantitative head-to-head comparison.

Thank you for suggesting this table. In the results section, we have added a qualitative summary table (Table 1) which highlights the triangulation across samples summary tables (Tables 3 & 4) for each analysis design to lay out overall quantitative results.

Minor comments:
Line 102 – the word "avuncular" is used here. I don't think this is the word you mean, since this refers to aunt-uncle / niece-nephew relationships.

Thank you for this feedback, which highlights that our description of the methods is unclear. These models do use aunt-uncle/niece-nephew relationships with MZ/DZ twins in the parent generation to decompose intergenerational associations into genetic and non-genetic factors. We have updated the text to add more clarification.

Introduction: *Line 122-132*
Briefly, the MCoTS design decomposes parent-child associations into genetic and environmental components by comparing monozygotic (MZ) and dizygotic (DZ) avuncular correlations (correlations between uncle/aunt and niece/nephew) to parent-child correlations. The children of MZ twins share 50% of their genes with their parent, and their parent's co-twin, meaning they are as genetically related to their aunt/uncle as they are to their parent, whereas children of DZ twins share 25% of their genes with their aunt/uncle. In this way, we can assess whether associations are stronger in more genetically related individuals to consider the role of genetics in intergenerational assessments, and whether there are remaining parent-child associations once controlling for shared genetics.

Supplemental Table 1: Consider either providing supplemental methods and/or a detailed footnote to interpret these parameters. The reader unfamiliar with SEM will not know how CFI, TLI, and RMSEA are similar/different to the p-values and regression model fit statistics they are used to seeing.

Supplemental Table 3: please indicate in the footnote what is meant by the bold font.

Thank you for flagging these missing notes from the supplementary tables. We have added the following text to the tables (now Supplementary Tables 4 & 12) footnote to support interpretation of the fit statistics: *Model fit indices were evaluated such that acceptable fit was determined when CFI > 0.90, the TLI > 0.90 and RMSEA < 0.08 (Hu & Bentler, 1999).*

We have also added the following text to the table (now Supplementary Table 6) footnote to clarify what is meant by the bold text: *Rows in **bold** are the final model, selected as the most parsimonious model.*

Reviewer #4 (Remarks on code availability):
Code was not provided, nor do I think that it is essential for this.
